# The chemical behavior of terminally *tert*-butylated polyolefins

**DOI:** 10.3762/bjoc.11.139

**Published:** 2015-07-24

**Authors:** Dagmar Klein, Henning Hopf, Peter G Jones, Ina Dix, Ralf Hänel

**Affiliations:** 1 Institut für Organische Chemie, Technische Universität Braunschweig, Hagenring 30, D-38106 Braunschweig, Germany, Fax: (+49)531-391-5388; 2 Current address: Mitsubishi Polyester Film GmbH, Kasteler Str. 45, 65203 Wiesbaden, Germany; 3 Institut für Anorganische und Analytische Chemie, Technische Universität Braunschweig, Postfach 3329, D-38106 Braunschweig, Germany, Fax: (+49)531-391-5387; 4 Current address: Novartis Pharma AG, Forum 1, Novartis Campus, CH-4056 Basel, Switzerland; 5 Current address: Bundesamt für Verbraucherschutz und Lebensmittelsicherheit (BVL), Messeweg 11/12, D-38104 Braunschweig, Germany

**Keywords:** bromination, Diels–Alder reactions, epoxidation, photochemistry, polyolefins, reactivity, hydrogenation

## Abstract

The chemical behavior of various oligoenes **2** has been studied. The catalytic hydrogenation of diene **3** yielded monoene **4**. Triene **7** was hydrogenated to diene **8**, monoene **9** and saturated hydrocarbon **10**. Bromine addition to **3** and **7** yielded the dibromides **17** and **18**, respectively, i.e., the oligoene system has been attacked at its terminal olefinic carbon atoms. Analogously, the higher vinylogs **19** and **20** yielded the 1,8- and 1,10-bromine adduts **23** and **24**, respectively, when less than 1 equivalent of bromine was employed. Treatment of tetraene **19** with excess bromine provided tetrabromide **25**. In epoxidation reactions, both with *meta*-chloroperbenzoic acid (MCPBA) and dimethyldioxirane (DMDO) two model oligoenes were studied: triene **7** and tetraene **19**. Whereas **7** furnished the rearrangement product **31** with MCPBA, it yielded the symmetrical epoxide **32** with DMDO. Analogously, **19** was converted to mono-epoxide **33** with MCPBA and to **34** with DMDO. Diels–Alder addition of **7** with *N*-phenyltriazolinedione (PTAD) did not take place. Extension of the conjugated π-system to the next higher vinylog, **19**, caused NPTD-addition to the symmetrical adduct **37** in good yield. Comparable results were observed on adding NPTD (equivalent amount) to pentaene **20** and hexaene **21**. Using **36** in excess provided the 2:1-adduct **40** from **21** and led to a complex mixture of adducts from heptaene **22**. With tetracyanoethylene (TCNE) as the dienophile, tetraolefin **19** yielded the symmetrical adduct **43**, although the reaction temperature had to be increased. Pentaene **20** and hexaene **21** led to corresponding results, adducts **44** and **45** being produced in acceptable yields. With nonaene **42** and TCNE the 2:1-adduct **48** was generated according to its spectroscopic data. Exploratory photochemical studies were carried out with tetraene **19** as the model compound. On irradiation this reacted with oxygen to the stable *endo*-peroxide **52**.

## Introduction

**Scheme 1:**
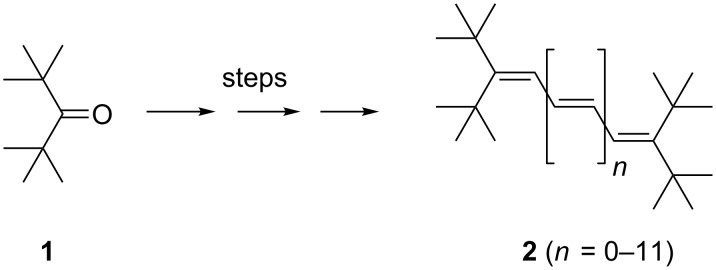
The polyenes **2** stabilized by terminal *tert*-butyl substituents.

Several years ago we described [2] a general synthesis of a series of terminally substituted, conjugated polyenes, **2**, beginning with the diene and ending with the decatriene (Scheme 1). We also reported the X-ray structures of compounds with *n* = 1, 2, 3, 4, 5 and 7 and discussed the structural similarities, in particular the distortions associated with the bulky *tert*-butyl groups.

For all these hydrocarbons, di-*tert*-butylketone (**1**) served as the starting material, which was chain-elongated by preparative sequences involving standard Wittig, Wittig–Horner and McMurry coupling reactions. Nearly all of the polyenes **2** (as well as many of their precursors) were characterized by X-ray structural analysis. These polyolefins are substructures of the most famous polyene, “polyacetylene”.

Having prepared and unambiguously characterized the hydro-carbons **2**, we now turn to their chemical behavior. In the sense that these polyolefins can serve as structural models for “polyacetylene”, their chemical properties should also reflect that of the polymer. Chemical reactions of the higher acyclic polyolefins have scarcely been studied, and we hence decided to carry out typical, textbook olefin reactions of a representative selection of the hydrocarbons **2**.

## Results and Discussion

### Catalytic hydrogenation

We started our studies on the reactive behavior of polyolefins **2** with one of the formally simplest alkene reactions: catalytic hydrogenation.

**Scheme 2:**
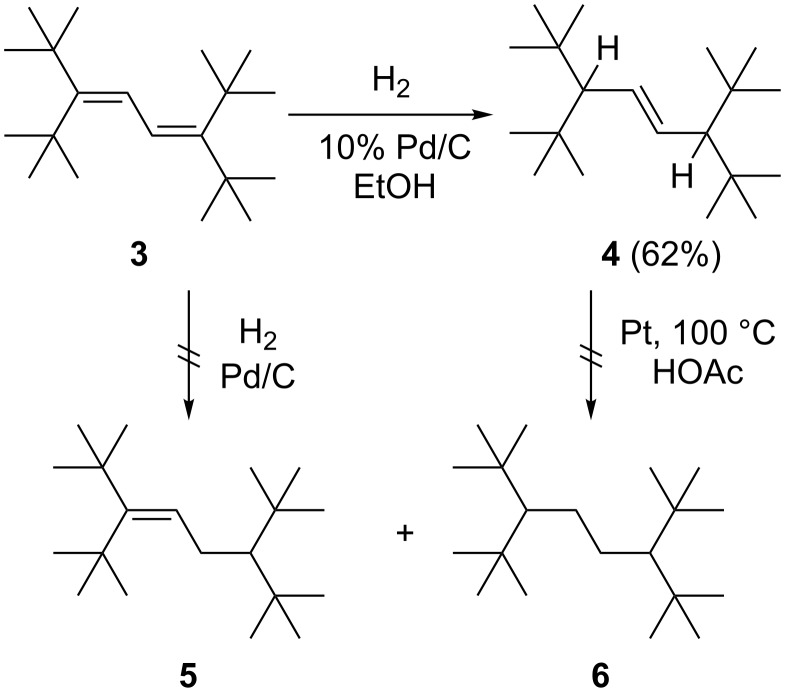
The catalytic hydrogenation of diene **3**.

When diene **3** was hydrogenated under relatively mild conditions (Pd/C, EtOH, room temp.), the only product that we could isolate was mono-olefin **4** (Scheme 2), a formal 1,4-addition product of hydrogen to **3**.

**Figure 1:**
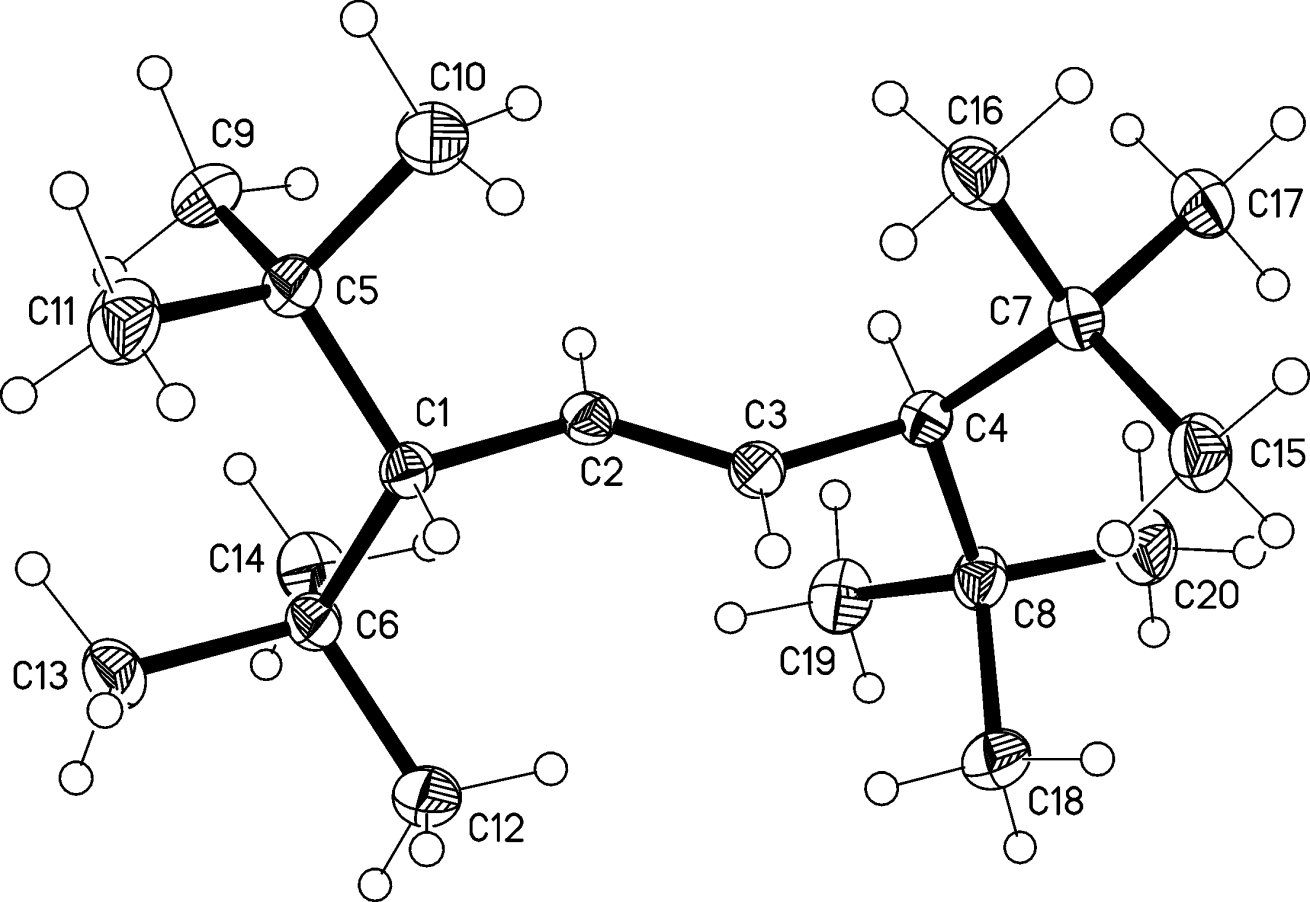
The structure of compound **4** in the crystal. Ellipsoids correspond to 30% probability levels.

Its structure was established by the usual spectroscopic and analytical data (see Supporting Information File 1). To determine the configuration of the double bond, an X-ray structural investigation was carried out. Although the quality of the structure determination was disappointing because of high residual electron density, it sufficed to determine the connectivity of the atoms in the compound and to demonstrate that the double bond configuration was *E* (Figure 1). The molecule possesses no crystallographic symmetry, but displays twofold symmetry to a good approximation (rms deviation 0.015 Å). The torsion angles across the central C1^…^C4 moiety are C5–C1^…^C4–C7 53.4° and C6–C1^…^C4–C8 −14.6°. Distortions from "normal" dimensions may be attributed to the steric effects of the *tert*-butyl groups; thus the C–CMe_3_ bonds are long (1.58 Å), the sp^3^ angles Me_3_C–C–CMe_3_ are wide (120°), and the sp^2^ C–C=C angles in the central chain are also wide (127°). For individual values, the deposited material should be consulted. The molecules pack end-to-end to form chains parallel to the *b* axis; neighboring chains extend the packing, again by translation, to layers parallel to the *bc* plane at *z* ≈ ¼ and *z* ≈ ¾.

The hydrogenation mixture yielded no evidence for the formation of a 1,2-adduct (**5**) or of the fully saturated hydrocarbon **6**. In fact, the primary adduct **4** was inert towards hydrogen even under very harsh hydrogenation conditions (Pt, 100 °C, acetic acid). We assume that the spatial shielding of the double bond, as indicated by the X-ray structure, is responsible for this. Furthermore, we were also unable to epoxidize or brominate **4**.

**Scheme 3:**
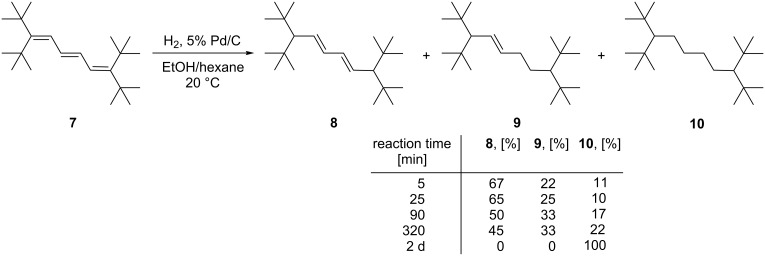
The catalytic hydrogenation of triene **7**.

The next higher vinylog of **3**, the triene **7**, is much more easily hydrogenated. Even under mild conditions (Pd/C, EtOH/hexane, room temp.) it is readily reduced (Scheme 3).

After 5 minutes of hydrogenation, three products were detected: the 1,6-adduct **8**, a diene (main product); the mono olefin **9**; and the fully saturated hydrocarbon **10**. When the reaction time was increased, the amounts of **9** and **10** grew at the cost of **8**, and when the hydrogenation was run for two days, alkane **10** was the only reduction product. It seems safe to assume that we are dealing with a stepwise process, with **8** and **9** serving as intermediates en route to **10**. Comparing the two experiments starting from **3** and **7**, it is obvious that two vicinal (*t*-Bu)_2_CH-substituents are required to shield a double bond from hydrogenation. The spectroscopic data of **8**–**10** can be found in Supporting Information File 1. Since we did not expect to obtain fundamentally new results with the oligoenes beyond **7**, we stopped the hydrogenation experiments at this stage.

Our hydrogenation results with α,ω-fully *tert*-butylated oligoenes are similar to the studies of Kuhn and Winterstein with several α,ω-diphenylpolyenes [3]. These authors hydrogenated their aromatic derivatives under different condi-tions; results comparable to ours were obtained with aluminum amalgam in moist ether. The primary product with the hexatriene, the octatetraene and the decapentaene derivatives were always the 1,ω-adducts. Although more highly reduced derivatives – similar to, e.g., **9** – were not obtained, exhaustive hydrogenation of the 1,8-hydrogenated products always provided the α,ω-diphenylalkanes.

### Brominations

**Scheme 4:**
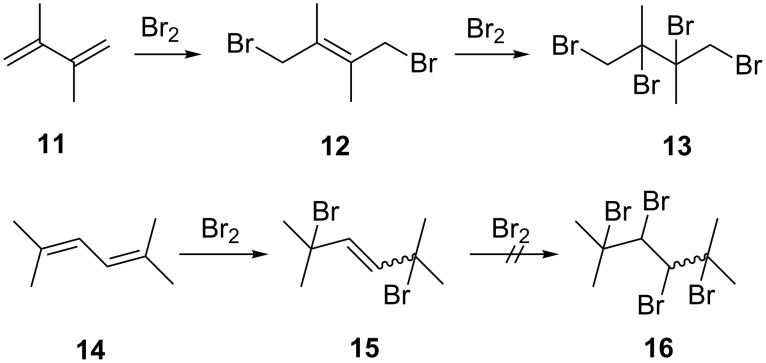
Addition of bromine to model dienes.

The behavior of dienes towards bromine has been studied extensively and, in particular, the addition of bromine to buta-1,3-diene is discussed in most textbooks on organic chemistry [4]. Elementary bromine adds to buta-1,3-diene in halogenated solvents to give mostly the 1,4-addition product (ratio 1,4-/1,2-product: 7:1) [5]. Turning to substituted dienes, 2,3-dimethylbuta-1,3-diene (**11**) initially also provides the 1,4-adduct **12**, which subsequently is saturated to the tetrabromide **13** by reaction with a second equivalent of bromine (Scheme 4) [6–8].

Whereas an isomer of **11**, hexa-2,5-diene, behaves similarly to **11** [9], the terminally fully methylated diene **14** does not react further than **15** on bromine addition, no tetrabromide **16** being formed [10].

**Scheme 5:**
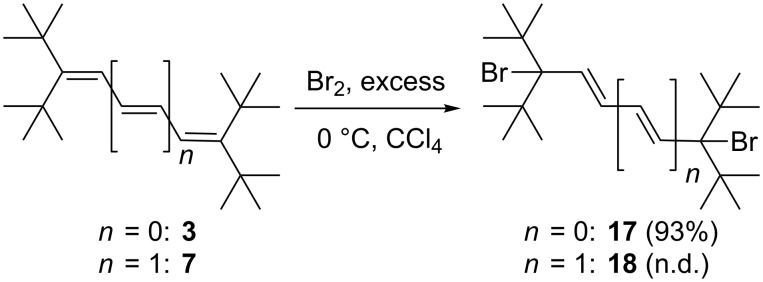
Bromine addition to diene **3** and triene **7**.

An increase in the steric bulk of the (terminal) substituents should lead to comparable results; and this is indeed the case (Scheme 5), as shown by diene **3**.

The 1,4-addition product **17**, a colorless solid, is produced in near quantitative yield (93%). It is easily characterized by its spectroscopic data, which are listed in Supporting Information File 1. Unfortunately, we were unable to prepare crystals of **17** suitable for an X-ray structural analysis.

Interestingly, Kuhn and Winterstein in their study on the chemical behavior of α,ω-diphenyloligoenes obtained different results: for 1,4-diphenylbuta-1,3-diene, bromine addition furnished the 1,2-adduct in 95% yield [3]. As in the case of the hydrogenation of **3**, 1,4-adduct **17** is inert to further bromine addition; the steric bulk of the substituents at the remaining double bond has increased even further.

When excess bromine is added to triene **7**, we also obtained only the 1,6-addition product **18**. This is inferred from the NMR spectra of the raw product mixture. The ^13^C NMR spectrum only displays 5 signals (see Supporting Information File 1), half the number of the signals expected for a less symmetrical 1,6-adduct than the one shown in Scheme 5. Again, no further adducts were detected, a fact which we ascribe to the “protection” of the double bonds by the (*t*-Bu)_2_CBr-substituents. Unfortunately, **18** is unstable. Even measurement of its NMR spectra is accompanied by decomposition: the color of the CDCl_3_ solution rapidly changed to brown, and then black. Again, this result is in marked contrast to Kuhn´s experiment with the 1,6-diphenylhexatriene. In this latter case tetra- and even hexabromo adducts were also obtained [3].

**Scheme 6:**
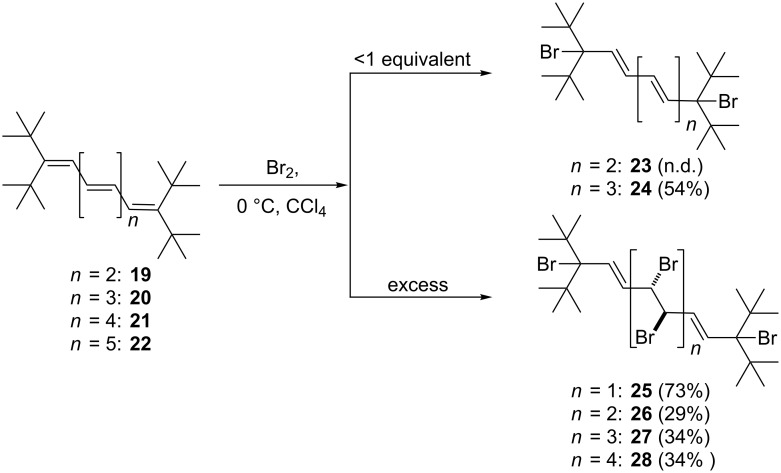
Bromine addition to the higher oligoenes **19–22**.

**Figure 2:**
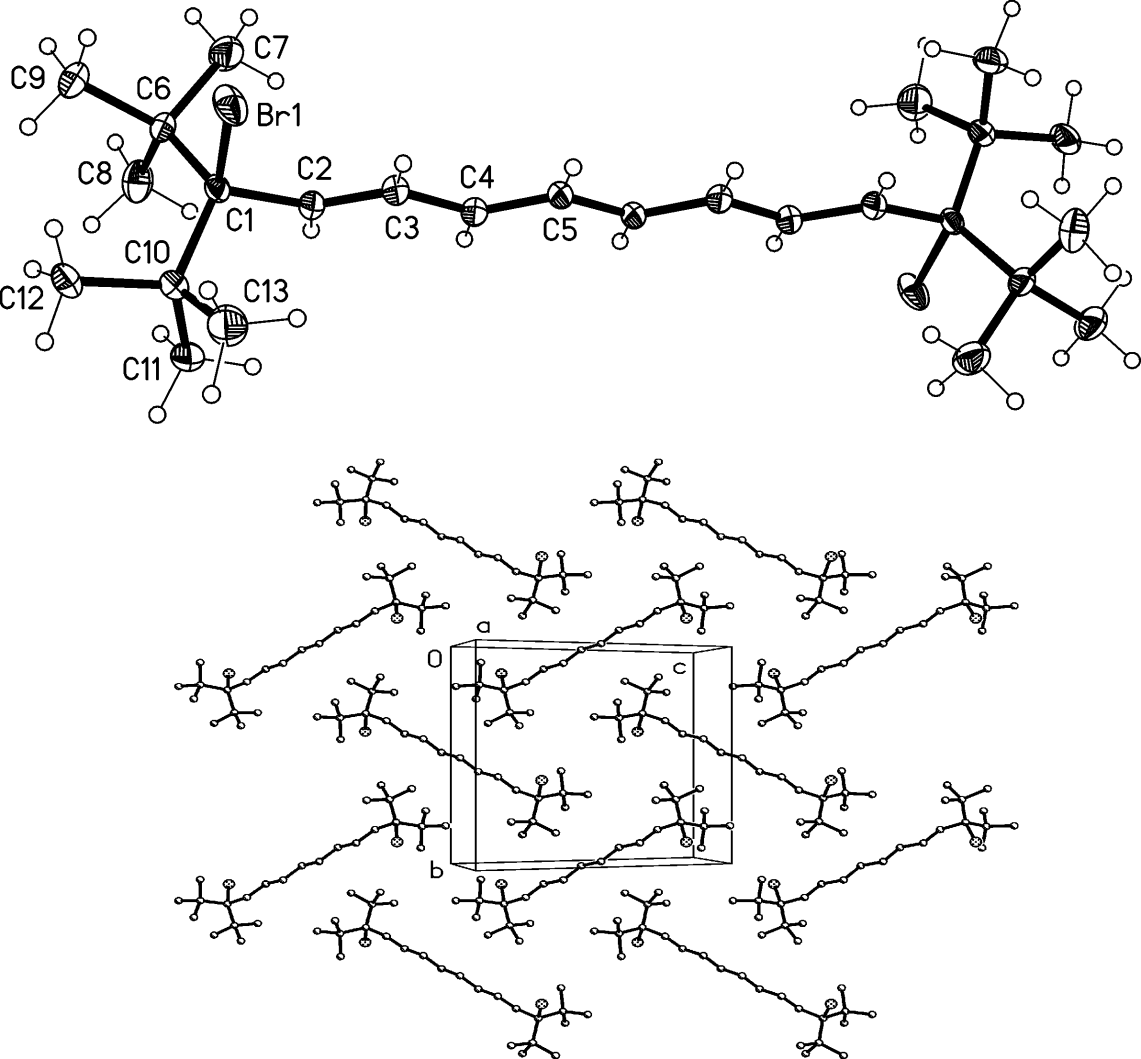
(a) The structure of compound **24** in the crystal. Ellipsoids correspond to 50% probability levels. (b) Packing diagram of compound **24** viewed perpendicular to (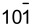). Hydrogen atoms are omitted.

Proceeding to the next higher vinylogs, **19** and **20**, an analogous reactivity pattern to that above is observed when less than an equivalent of bromine is employed. However, in the case of **19** only a product mixture containing the 1,8-adduct **23** as the minor component (**19**:**23** 2.5:1) was obtained, from which the (unstable) **23** could not be isolated in pure form (Scheme 6). In the case of **20** we were more successful: the dibromide **24** was not only obtained in analytically pure form, but also as colorless needles suitable for an X-ray investigation. Whereas the spectroscopic data of **23** and **24** are given in Supporting Information File 1, the structure of **24** in the solid state is discussed here. The molecule (Figure 2a) possesses exact inversion symmetry, but its non-crystallographic symmetry is close to 2/*m* (*C*_2_*_h_*), with an rms deviation of 0.21 Å. Distortions associated with the *tert*-butyl groups are largely similar to those discussed above for **4**, but the angle C1–C2=C3 is wider still at 131° (other chain angles are 122–125°). The bromine atom is approximately synperiplanar to C3 across the C1–C2 bond (torsion angle 12.2°). The molecular packing (Figure 2b) involves herringbone-type layers perpendicular to (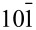). The shortest H^…^Br contacts of 3.05 Å, probably of only marginal significance, are formed between layers.

Having unambiguously established that the mono adduct of bromine to pentaene **20** has the structure of a 1,10-addition product with all-*E*-configuration of the double bonds, i.e., **24**, we postulate that the corresponding adducts, **17**, **18** and **23**, have analogous structures, as shown in Scheme 5 and Scheme 6, respectively.

**Figure 3:**
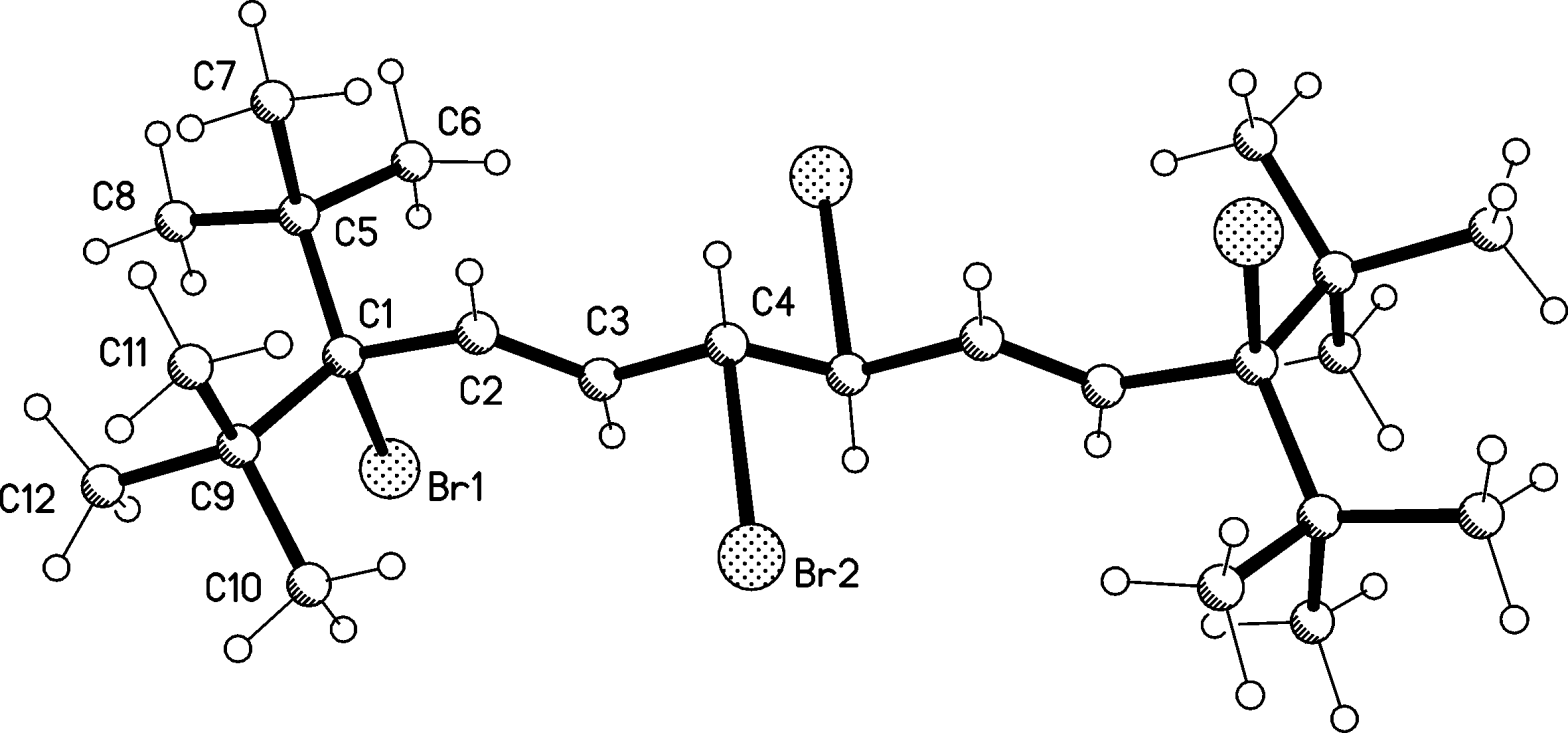
The structure of compound **25** in the crystal. This was a structure of poor quality and served only to determine the connectivity.

When the tetraene **19** was treated with a six-fold excess of bromine, a tetrabromide **25** was produced in good yield (73%). The compound could be kept at −10 °C without decomposition for considerable times and was recrystallized from pentane to provide needles "suitable" for X-ray structural analysis; its qualitative structure in the solid state is reproduced in Figure 3, but should be interpreted with great caution because of unsatisfactory refinement. The compound appeared to crystallize in *P*2_1_/*c* with *a* = 10.243, *b* = 10.467, *c* = 13.222 Å, β = 96.39° and *Z* = 2 (at −130 °C). The refinement was unsatisfactory because the carbon atoms could not be refined anisotropically, and several bond lengths and angles were unrealistic. Possible sources of error would include disorder, unidentified weak reflections corresponding to a larger cell (the data were recorded on a serial diffractometer), or an incorrect space group. Refinements in lower symmetry space groups were, however, not better. We believe that the chemical nature of compound **25** has nevertheless been qualitatively confirmed.

The spectroscopic data of **25** (see Supporting Information File 1) are consistent with its solid state structure. There are several plausible mechanisms that would explain the formation of **25**. This bis-bromine adduct could be formed from **19** either by two consecutive 1,4-bromine addition processes or by a 1,8-addition followed by *trans*-bromination of its central double bond. This bond should be favored for steric reasons over the two terminal double bonds of the triene intermediate **23**. Since this dibromide was not available in pure form, we did not carry out the control experiment required to distinguish experimentally between the first and the second path.

The higher oligoenes **20**, **21**, and **22** react similarly to **19** with excess bromine and provide the corresponding polybromides **26–28** in the yields shown in Scheme 6. Since we were not able to obtain X-ray data for these adducts, their exact stereostructures must be left open for the time being. It is quite clear, though, that the terminal, highly substituted quaternary carbon atoms protect their neighboring double bond from further attack. The attack by “positive” bromine at the di-*tert*-butylated carbon atoms in the first step of the addition process is probably associated with the better stabilization by resonance of the cationic intermediate thus generated compared to the alternative positively charged intermediate produced by initial bromine attack at the penultimate carbon atom. In the former case the positive charge can shift to the other end of the polyolefin system, where it is stabilized by the combined hyperconjugative effect of two *tert*-butyl groups. It is this other terminal carbon atom that is attacked by the bromide ion in the termination step, resulting in the overall formation of 1,x-bromine adducts.

### Epoxidations

**Scheme 7:**
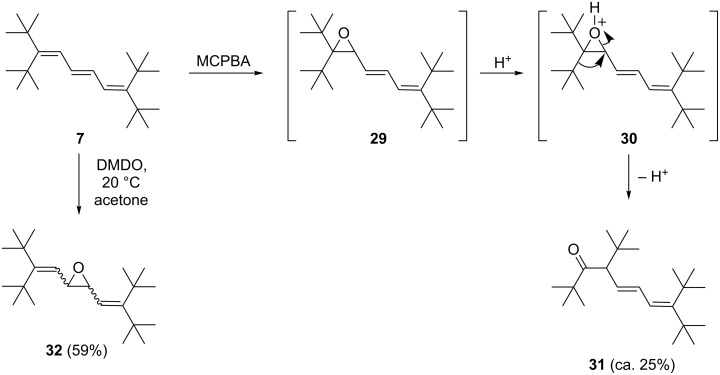
Epoxidation of triene **7** with MCPBA and DMDO.

**Scheme 8:**
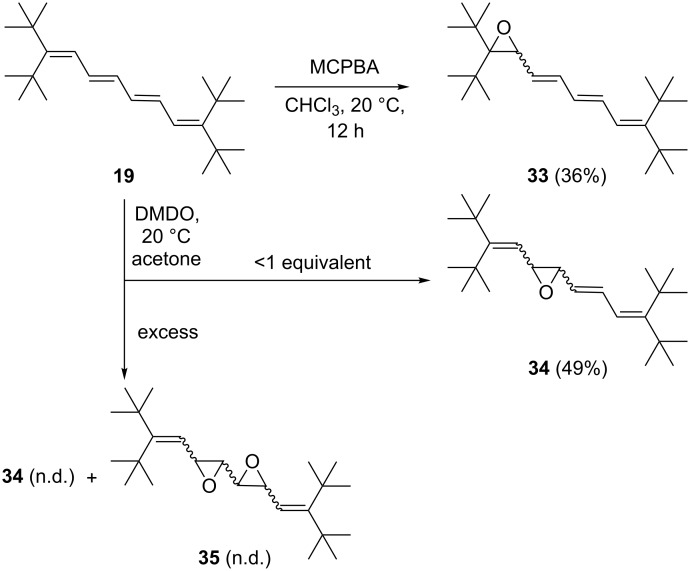
Epoxidation of tetraene **19** with MCPBA and DMDO.

Epoxidation reactions of highly substituted dienes have been carried out by us and by others, and it has been demonstrated that whenever competing reaction pathways exist, it is usually the more highly substituted double bond that is preferentially attacked, regardless of the steric bulk of the substituents [11]. As far as the higher vinylogs are concerned, we decided to study an odd oligoene, the triene **7** (Scheme 7), and an even representative, the tetraene **19** (Scheme 8).

In the first epoxidation experiment, **7** was treated with *m*-chloroperbenzoic acid (MCPBA) in chloroform at room temperature overnight. Surprisingly, it was neither the mono- epoxide **29** nor its non-terminal isomer **32** but the ketone **31** that was obtained in low yield (ca. 25%). The structure follows from the spectroscopic data (see Supporting Information File 1); the carbonyl group is readily seen in the IR spectrum (ν_max_ = 1702 cm^−1^) and the carbonyl carbon signal in the ^13^C NMR spectrum at δ = 217 ppm is also of particular diagnostic value. We propose that **31** is produced from epoxide **29** by initial protonation to the oxonium ion **30**, which then undergoes a Wagner–Meerwein rearrangement followed by deprotonation. Under non-acidic conditions, this process would not be expected; and indeed, when **7** was oxidized with dimethyldioxirane (DMDO) in acetone at room temperature, epoxide **32** with a central oxirane ring is produced in acceptable yield (59%). Since we were unable to obtain single crystals of this derivative, the assignment of its exact stereostructure (*syn*- or *anti*-epoxide) must remain tentative. Since many other epoxidations take place with retention of the original double bond configuration, we assume that the *anti*-configuration is more probable in the present case as well. The spectroscopic data (see Supporting Information File 1) also support the structure shown in Scheme 7. Of particular value is the ^13^C NMR spectrum, which shows a halved set of signals, as expected for a symmetrical structure. The completely substituted olefinic carbon atoms display a signal at δ = 161 ppm, whereas the –CH= carbon atoms absorb at 122 ppm. The oxirane carbon atoms appear at 59 ppm.

When the double bond chain is extended by one –CH=CH– group, results were obtained comparable to those observed for **7** (Scheme 8). Thus, tetraene **19** yielded mono-epoxide **33** with MCPBA in chloroform in fair yield (36%). With DMDO in sub-stoichiometric amounts, mono-epoxide **34** was produced in acceptable yield (49%). Employing excess DMDO yields a complex product mixture in which we could identify **34** as well as more highly epoxidized products (by mass spectrometric analysis) to which we tentatively assign structures **35**. Our attempts to obtain pure products by chromatographic separation have in this latter case so far failed.

To summarize the epoxidation experiments, it appears that MCPBA prefers to attack the terminal double bonds of our sterically shielded oligoenes, whereas there are indications that DMDO oxidizes “inner” double bonds preferentially. In principle, heterocycles with larger rings could also be produced in these experiments; however, at present we have no experimental evidence for these alternative routes.

### Diels–Alder reactions

**Scheme 9:**
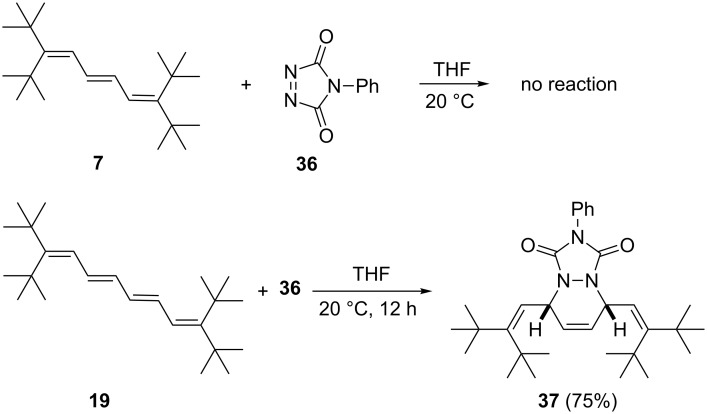
Diels–Alder addition of PTAD (**36**) to triene **7** and tetraene **19**.

Since we expected steric hindrance effects to play a pronounced role in the Diels–Alder additions of the shielded oligoene **3**, we decided to begin our experiments with one of the most reactive dienophiles, *N*-phenyltriazolinedione (**36**, PTAD, Scheme 9).

No reaction took place between triene **7** and **36**, even if the reaction mixture was heated to reflux. Clearly, the steric influence of the four bulky substituents is too great, and it either prevents the population of a cisoid conformation of diene **7** or it causes too much steric hindrance between the two cycloaddition partners en route to the transition state. This situation changes when the polyene chain is elongated by one double bond: tetraene **19** and dienophile **36** provide cycloadduct **37** in good yield (75%) under mild reaction conditions. Note that only this symmetrical regioisomer is produced – the alternative involving a terminal diene unit is not observed. The spectroscopic data (see Supporting Information File 1) already hinted that the cycloadduct **37** had been produced; the structure assignment was confirmed by X-ray structural analysis.

**Figure 4:**
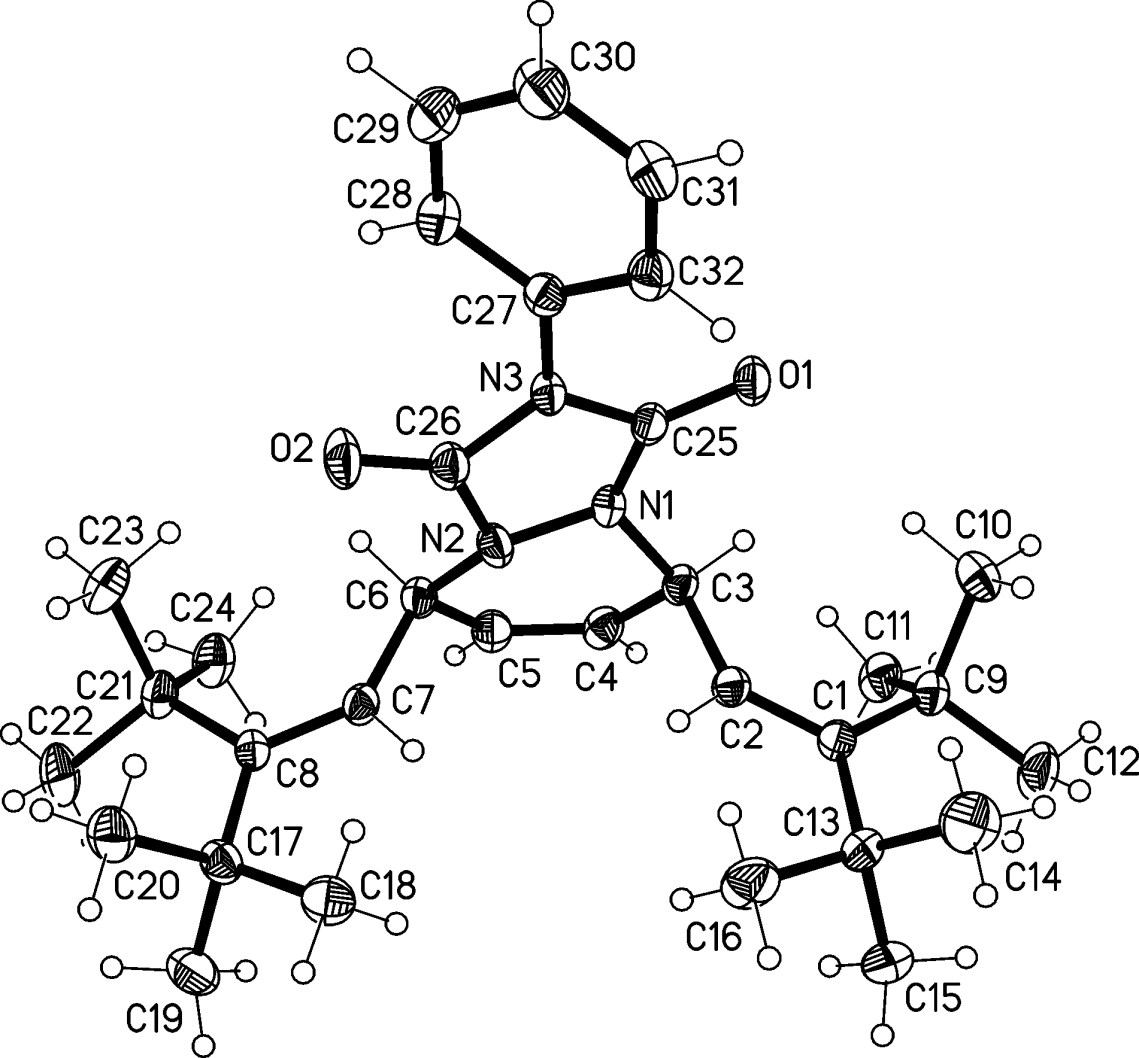
The structure of compound **37** in the crystal. Only one of two independent molecules is shown. Ellipsoids correspond to 30% probability levels.

The structure of **37** involves two independent molecules, one of which is shown in Figure 4. The molecules are closely similar, with an rms deviation of only 0.09 Å for all non-H atoms. The angles at C2 and C7 are greatly widened (to 132–135°). The central six-membered ring displays a 1,2-diplanar ("sofa") conformation, whereby the atom N1 lies 0.5 Å out of the plane of the other five atoms.

**Scheme 10:**
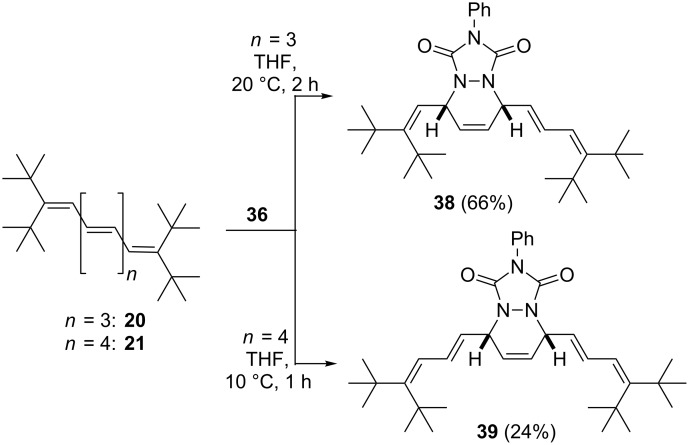
Diels-Alder addition of oligoenes **20** and **21** with PTAD (**36**).

The next two higher vinylogs, pentaene **20** and hexaene **21**, reacted similarly with PTAD (Scheme 10).

In neither case are the terminal double bonds involved in the cycloaddition process. In the case of pentaene **20**, the unsymmetrical adduct **38** was isolated in 66% yield, and with hexaene **21** the symmetrical adduct **39** was obtained. The lower yield (24%) in this case is misleading, since we also re-isolated 50% of the starting oligoolefin **21** in this experiment with just one equivalent of PTAD. The course of the reaction in the latter case is more difficult to follow. Whereas **37** and **38** are colorless, cycloadduct **39** is yellow. In the former cases the reactions can be followed visually analogous to a titration, with the intensely colored PTAD serving as the indicator. In case of the transformation **21**→**38**, the color changes from red to yellow and is hence less readily monitored.

**Scheme 11:**
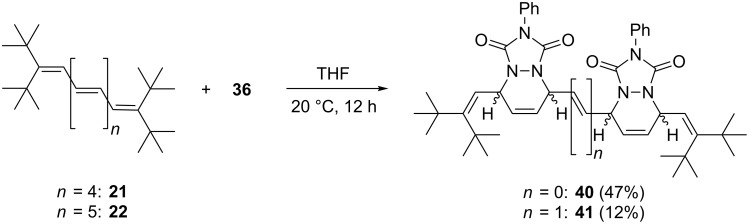
Addition of excess PTAD (**36**) to hexaene **21** and heptaene **22**.

When an excess of **36** is employed in the Diels–Alder reaction, the overall picture changes (Scheme 11).

With **21** (and extended reaction times) the 2:1-adduct **40** is produced in acceptable yield (47%) as the main product. In other words, both penultimate double bonds have also participated in the addition process (again the terminal double bonds remain untouched). Very probably this process also occurs stereospecifically, but since we could not obtain single crystals of X-ray quality in this case we refrain from stereochemical assignments (note also that the added heterocycles could in principle be in *syn-* or *anti*-orientation).

Chemically and stereochemically the situation becomes even more intricate on further extension of the polyene chain. When heptaene **22** is treated with an equimolar amount of **36**, a complex mixture is obtained consisting of starting material, mono-adducts (25%, MS analysis) and bis-adducts (12%). Chromatographic separation of the products turned out to be impossible, but we believe that compound **41** shown in Scheme 11 is among them; as in all other experiments the terminal double bonds remained untouched.

**Scheme 12:**
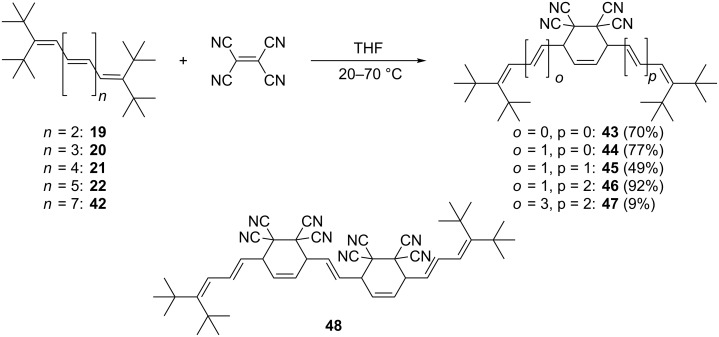
TCNE addition to oligoolefins: from tetraene **19** to nonaene **42**.

Tetracyanoethylene (TCNE) is usually less reactive as a dienophile than PTAD; this is also the case when the above mentioned oligoenes are employed as the diene components (Scheme 12). As expected, triene **7** did not react with TCNE, neither at room temperature nor at elevated temperatures. Tetraene **19** gives the expected (see Supporting Information File 1 for experimental details) symmetrical adduct **43**, but only under reflux conditions (THF, 65 °C). In addition to the spectroscopic data, the solid state structure of **43** was determined by crystal structure analysis.

**Figure 5:**
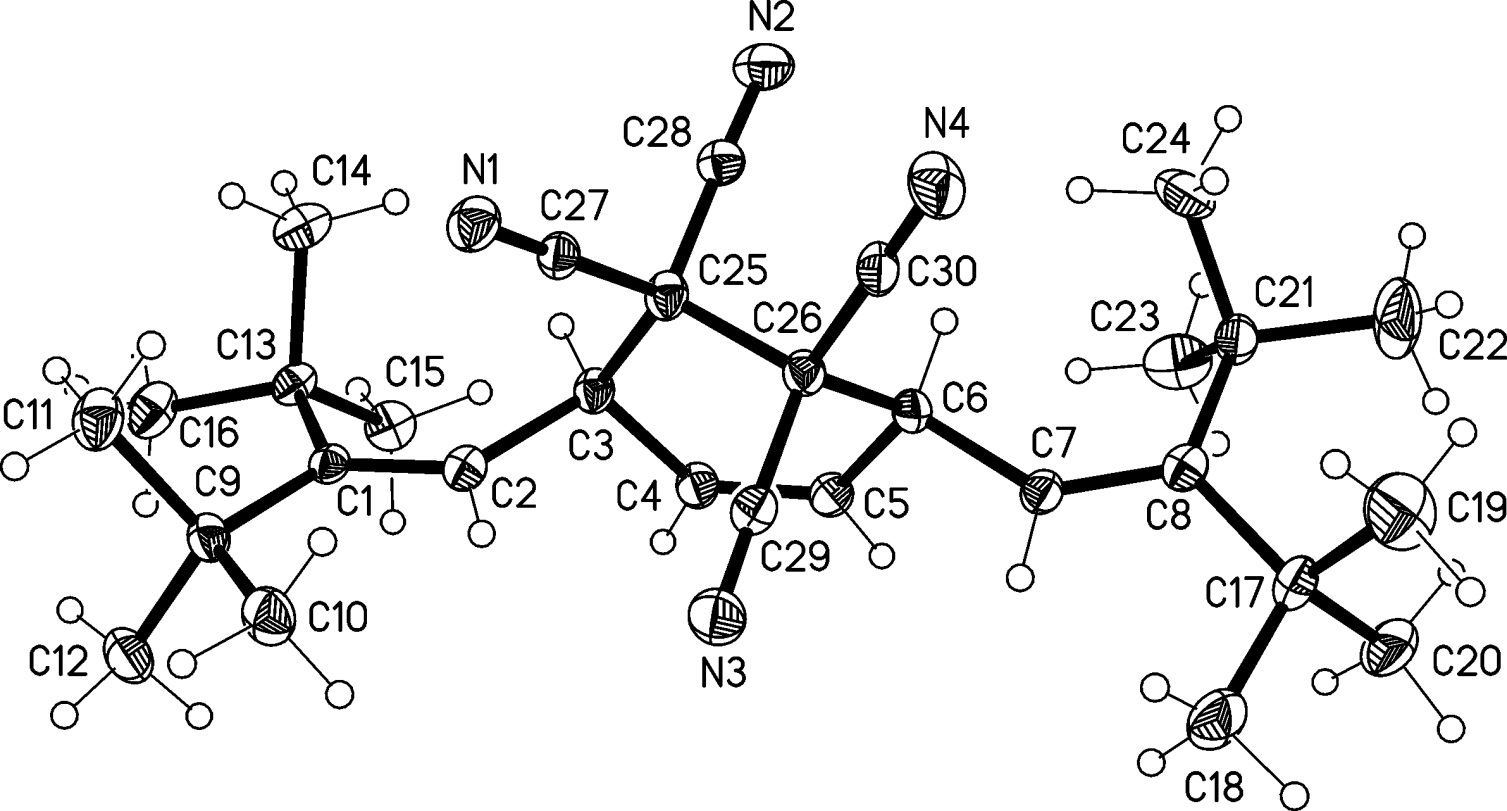
The structure of compound **43** in the crystal. Only one of two independent molecules is shown. Ellipsoids correspond to 30% probability levels.

The structure of this adduct is in many ways similar to that of the related PTAD-adduct **37**. The ring conformation is again a "sofa", whereby C26 lies 0.7 Å out of the plane of the other five atoms (Figure 5). The angles at C2 and C7 are again wide at 131–132°, although not quite as wide as for **37**. By chance, there are again two independent molecules in the asymmetric unit that are closely similar (the rms deviation for all non-H atoms except methyl C is 0.08 Å).

As expected, from pentaene **20** the Diels–Alder adduct **44** is produced in 77% yield, and from hexaene **21** the mono-adduct **45** (49%). Finally, with heptaene **22** and nonaene **42** the adducts **46** and **47** were obtained in varying yields. In the latter case some decomposition of the product was noted during work-up and we isolated a TCNE bis-adduct, **48**, for the first time in this series (Scheme 12). Again, we do not attempt to describe the exact stereochemical outcome of the addition, since the experimental evidence is too meagre.

Exploratory cycloaddition experiments were carried out with dimethyl acetylenedicarboxylate and maleic anhydride, but none of these dienophiles reacted with, e.g., heptaene **22** up to 75 °C.

In summary, the *tert*-butyl protected oligoenes participate in Diels−Alder reactions, as diene components with up to nine consecutive double bonds, with very reactive dienophiles (PTAD, TCNE). Only in the most extended cases are 2:1 adducts produced, and in all cases the terminal double bonds survive the cycloaddition process.

### Photochemical behavior

Although oligo- and polyene substructures are present as chromophores in, inter alia, the visual pigments [12], the carotenoid antennae of photosynthesis [13–14], and vitamins A [15] and D [16], relatively little is known about their basic photochemical reactions, such as photoisomerizations and/or photoadditions. For the unsubstituted hydrocarbons, this is not surprising in view of their general instability and the difficulty of obtaining pure diastereomers.

**Scheme 13:**
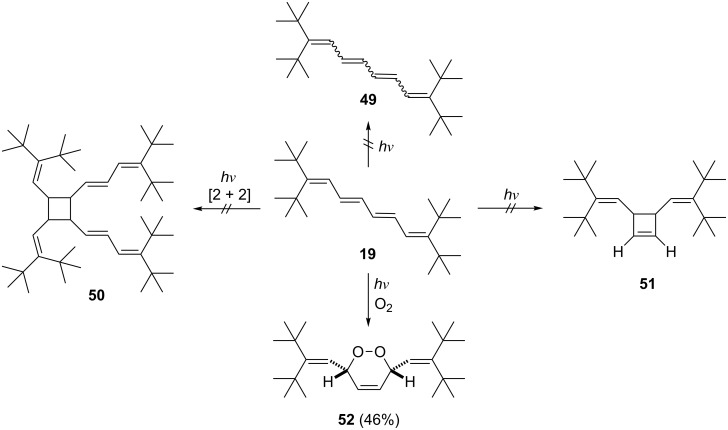
Photochemical experiments with tetraene **19**.

With our stabilized oligoenes in hand, we started an exploratory study to investigate typical photoreactions of unsaturated systems. As illustrated in Scheme 13, we selected tetraene **19** as a model compound, since the previous investigations had shown (see above) that in many cases four consecutive double bonds are required to observe chemical transformations.

The first two processes involved photoisomerizations. It is well known that *E*/*Z*-isomerizations take place readily in oligoenes, even when daylight is used for photoexcitation. However, rather than isomerizing to **49**, a mixture of diastereomers, **19** remained unchanged when irradiated with a daylight lamp in deuteriochloroform. The only process that we could observe was a “polymerization” reaction, which slowly destroyed the substrate. Likewise, no photocyclization to the divinylcyclobutene derivate **51** was noted. [2 + 2] Photodimerizations of olefins have often been described, whether in solution or in the solid state. For **19**, this reaction, which would lead to the photodimer **50** – or any other cycloadduct – was not observed. It is known from the solid state structures of the *tert*-butylated oligoenes that the distance between two polyolefin chains is markedly larger than the intermolecular distance between two double bonds that successfully undergo a [2 + 2] cycloaddition (between 3.5 and 4 Å, the so-called “topochemical reaction control”). Furthermore, in derivatives **2** two adjacent polyolefin chains are orthogonal to each other, because of the steric bulk of the *tert*-butyl moieties. The p-orbitals of the double bonds are hence prevented from overlap [1–2].

So far we have only been successful in observing a single photochemical reaction between **19** and an “external” reagent: the photoaddition of oxygen to its unhindered, inner diene unit. After 20 h irradiation with a daylight lamp in deuteriochloroform solution in the presence of air, *endo*-peroxide **52** was isolated in 46% yield. Its structure follows from its spectroscopic data (see Supporting Information File 1) and, in particular, an X-ray structural investigation of single crystals obtained from a petrol ether solution.

**Figure 6:**
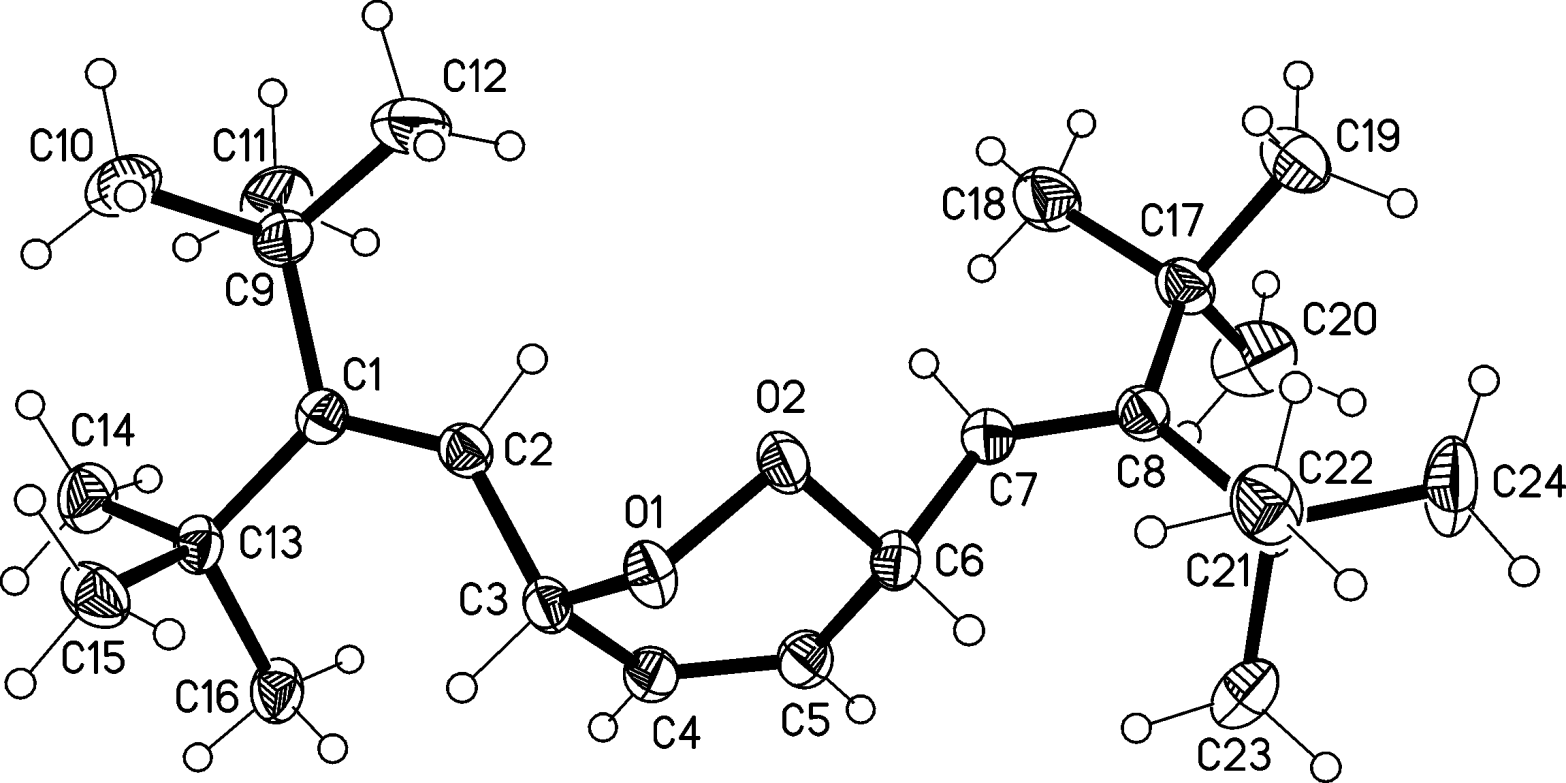
The structure of compound **52** in the crystal. Ellipsoids correspond to 50% probability levels.

The molecule of **52** is shown in Figure 6. The O–O bond length is 1.4755(12) Å, which corresponds well to the mean value of 1.480 Å obtained from the Cambridge Crystallographic Database [17] for similar ring systems (69 hits, 81 values; one severe outlier omitted). The six-membered ring has an approximate “sofa” conformation, whereby O1 lies 0.75 Å out of the plane of the other five atoms. The angles at C2 and C7 are 134, 132°. Despite the steric shielding provided by the *tert*-butyl groups, the molecules associate in pairs via a weak hydrogen bond H3^…^O1, 2.57 Å.

The photochemical addition of oxygen to numerous diene systems has been investigated by many authors [18–20]. In most cases this photooxidation involves singlet oxygen that is generated from triplet oxygen by irradiation in the presence of a sensitizer such as chlorophyll. Since the above experiment was carried out in the absence of a sensitizer, the formation of **52** must be explained by a different mechanism. One alternative could be the photochemical generation of a diradical from the conjugated oligoene **19** and interception of the former by the oxygen present in the reaction solution. Interestingly, when the solution is degassed before irradiation and the photolysis is carried out under argon, only polymeric material is produced from **19** after extended irradiation (20 h).

## Supporting Information

File 1 Experimental part.
