# Supplementary material for: The chemical behavior of terminally tert-butylated polyolefins
Source: Beilstein J Org Chem. 2015 Jul 24;11:1246–58. doi: 10.3762/bjoc.11.139 (PMC4578339; doi:10.3762/bjoc.11.139)
Supplement: File 1 — Experimental part. [file Beilstein_J_Org_Chem-11-1246-s001.pdf]

Supporting Information

for

The chemical behavior of terminally *tert*-butylated polyolefins

Dagmar Klein[1,2], Henning Hopf*[1], Peter G. Jones[3], Ina Dix[1,4] and Ralf Hänel[1,5]

Address: [1]Institut für Organische Chemie, Technische Universität Braunschweig, Hagenring 30, D-38106 Braunschweig, Germany, Fax: (+49)531-391-5388, [2]Current address: Mitsubishi Polyester Film GmbH, Kasteler Str. 45, 65203 Wiesbaden, Germany, [3]Institut für Anorganische und Analytische Chemie, Technische Universität Braunschweig, Postfach 3329, D-38106 Braunschweig, Germany, Fax: (+49)531-391-5387, [4]Current address: Novartis Pharma AG, Forum 1, Novartis Campus, CH-4056 Basel, Switzerland and [5]Current address: Bundesamt für Verbraucherschutz und Lebensmittelsicherheit (BVL), Messeweg 11/12, D-38104 Braunschweig, Germany

E-mail: Henning Hopf – h.hopf@tu-bs.de

*Corresponding author

Experimental part

General remarks:

Chromatography: TLC: Polygram Sil G/$UV_{254,}$ Macherey, Nagel & Co. (Düren). – CC: Kieselgel 60 (70-230 mesh), Merck. – M.p.: <200 °C: Büchi 510, >200 °C: Kofler "Thermopan", Reichert (Vienna); the melting points are uncorrected. – NMR: ^{1}H- and ^{13}C-spectra: Bruker AC-200, ^{1}H NMR: 200.1 MHz, ^{13}C NMR: 50.3 MHz; Bruker DRX-400, ^{1}H NMR: 400.1 MHz, ^{13}C NMR: 100.6 MHz; int. standard: TMS; spin multiplicities of ^{13}C signals: DEPT-technique. – IR: solids: KBr pellets, liquids: film, NICOLET 320 FTIR-spectrometer; ATR-IR-spectra: Bruker Tensor 27. – UV-vis: HP 8452A Diode Array or Varian Cary 100 BIO. – MS: FINNIGAN MAT 90x, EI: 70 eV or CI (ammonia). GC/MS: FINNIGAN TSQ 700 (EI, 70 eV) coupled to a HP 5890A GC. - Dimethyldioxirane (DMDO) was prepared according to ref. [1].

Hydogenation reactions:

Hydrogenation of diene **3**: A hydrocarbon mixture (200 mg) containing triene **3** (112 mg, 0.40 mmol) was dissolved in ethanol (20 mL) and hydrogenated using 10% Pd on charcoal (5 mg) as the catalyst. The catalyst was removed by filtration and the solvent evaporated by rotary evaporation. The residue was taken up in diethyl ether and the solution subjected to preparative gas chromatography (Carbowax, 3 m, 160 °C). The main fraction (69 mg, 62%) consisted of the 1,4-addition product **4**, colorless crystals, m.p. 97-98 °C. – ^{1}H NMR (400.1

MHz, $CDCl_3$): δ = 1.00 (s, 36 H, *tert*-Bu), 1.61 (AA´XX´, 2 H, 3-, 6-H), 5.23 ppm (AA´XX´, 2 H, 4-, 5-H). - ^{13}C NMR (100.6 MHz, $CDCl_3$): δ = 132.6 (d, C-4, -5), 62.2 (d, C-3, -6), 35.4 (s, C-3, -7), 31.3 ppm (q, *tert*-Bu). – IR (KBr): nu(tilde) = 3012 (vs), 1394 and 1364 cm^{-1} (m, *tert*-Bu). – UV (acetonitrile): λ_{max} (lg ε) = 192 nm (3.99). – $C_{20}H_{40}$ (280.54): calcd.: C 85.63, H 14.37; found: C 85.53, H 14.40.

<u>Total hydrogenation of triene **7**:</u> To a solution of triene **7** (50.0 mg, 0.16 mmol) in a mixture of ethanol (15 mL) and hexane (5 mL) was added ca. 30 mg of 5% palladium on charcoal and the mixture hydrogenated at room temp. for 2 d. The catalyst was removed by filtration, the solvents were evaporated by rotary evaporation and the residue chromatographed on silica gel with pentane, yielding 43 mg (85%, isolated yield of only product) of the colorless hydrocarbon **10**, m.p. 65 °C. - ^1H NMR (400.1 MHz, $CDCl_3$): δ = 0.80-0.82 (m, 2 H, 3-, 8-H), 0.98 (s, 36 H, *tert*-Bu), 1.23-1.26 (m, 4 H, 4-, 7-H), 1.28-1.31 ppm (m, 4 H, 5-, 6-H). – ^{13}C NMR (100.6 MHz, $CDCl_3$): δ = 58.37 (d, C-3, -8), 36.71 (s, C-2, -9, -11, -13), 35.11 (t, C-5, -6), 30.88 (q, C-1, -10, -12, -14), 30.19 ppm (t, C-4, -7). - IR (KBr): nu(tilde) = 2959 (vs), 2860 (s), 1489 (s), 1391, 1374 (*tert*-Bu, m), 719 cm^{-1} (w). - UV (acetonitrile): no absorption maxima between 190 and 820 nm. - MS (70 eV): *m/z* (%) = 252 (8) [M^+-*tert*-Bu], 196 (20) [M^+- 2 x *tert*-Bu], 183 (4), 169 (6), 113 (8), 57 (100). –

elemental analysis: calcd.: C 85.07, H 14.93; found: C 84.57, H 15.24.

Partial hydrogenation of triene **7**: To a solution of triene **7** (50 mg, 0.16 mmol) in a mixture of ethanol (20 mL) and hexane (6 mL) was added ca. 30 mg of 5% palladium on charcoal and the mixture stirred for various times (see Table in Scheme 3). Work-up according to the procedure given above yielded, for example after 90 min, a product mixture containing 50% of **8**, 33% of **9** and 17% of **10** (GC analysis). The individual NMR spectra of **8** and **9** can be derived from the spectrum of the hydrogenation mixture; the mass spectra of these compounds were determined by a GC/MS investigation. – Spectroscopic and analytical data of **8**: ^{1}H NMR (400.1 MHz, $CDCl_3$): δ = 0.97 (s, 36 H, *tert*-Bu), 1.58 (d, ^{3}J = 10.59 Hz, 2 H, 3-, 8-H), 5.47-5.53 (AA´XX, 2 H, 4-, 7-H), 5.78-5.82 ppm (AA´XX´, 2 H, 5-, 6-H). - ^{13}C NMR (100.6 MHz, $CDCl_3$): δ = 132.78 (d, C-4, -7), 131.78 (d, C-5, -6), 62.02 (d, C-3, -8), 35.05 ppm (s, C-2, -9, -11, -13). - MS (FD): *m/z* = 306.6. - MS (GC/MS): *m/z* (%) = 306 (5) [M^+], 249 (13) [M^+-*tert*-Bu], 193 (25) [M^+-2 x *tert*-Bu], 125 (45), 57 (100). – Spectroscopic and analytical data of **9**: ^{1}H NMR (400.1 MHz, $CDCl_3$): δ = 0.86-0.87 (m, 1 H, 8-H), 0.97, 0.99 (s, 36 H, *tert*-Bu), 1.28-1.34 (m, 1 H, 7-H), 1.52 (d, ^{3}J = 10.33 Hz, 1 H, 3-H), 2.03-2.05 (m, 1 H, 6-H), 5.14-5.21 (dt, ^{3}J =6.74, ^{3}J = 13.48 Hz, ^{3}J = 15.12 Hz, 1 H, 5-H), 5.32-5.38 ppm (dd, ^{3}J = 10.34, ^{3}J = 15.07 Hz, 1 H, 4-H). - ^{13}C NMR (100.6 MHz, $CDCl_3$): δ = 131.38 (d, C-5), 130.71 (d, C-4), 61.70 (d, C-3),

57.72 (d, C-8), 37.22 (t, C-6), 36.72, 34.70 (C-2, -9, -11, -13), 31.03 (q, C-1, -10, -12, -14), 30.27 ppm (t, C-7). - MS (FD): *m/z* = 308.6. - MS (GC/MS): *m/z* (%) = 308 (6) [M^+], 252 (11), 196 (25) 125 (21), 57 (100).

Bromine additions:

Br_2 addition to diene **3**: To a solution of diene **3** (50.0 mg, 0.18 mmol) in carbon tetrachloride (10 mL) was added at 0 °C a solution of bromine (300.0 mg, 1.88 mmol) in carbon tetrachloride (20 mL). After stirring the reaction mixture at this temperature, the solution was left at room temp. overnight. For work-up, the mixture was washed with aqueous $Na_2S_2O_5$-solution and the organic phase dried with sodium sulfate. Solvent removal by rotary evaporation at room temp. yielded the adduct **17** (73.0 mg, 93%) as a colorless solid, m.p. 104 °C. - ^1H NMR (400.1 MHz, $CDCl_3$): δ = 1.28 (s, 36 H, *tert*-Bu), 6.14 ppm (s, 2 H, 4-, 5-H). - ^{13}C NMR (100.6 MHz, $CDCl_3$): δ = 134.87 (d, C-4, -5), 98.03 (s, C-3, -6), 44.18 (s, C-2, -7, -9, -11), 31.16 ppm (q, C-1, -8, -10, -12). - IR (KBr): nu(tilde) = 2964 (vs), 1394, 1370 (*tert*-Bu, w), 985 (m), 595 cm^{-1} (C-Br, w). - UV (acetonitrile): λ_{max} (lg ε) = 214 (4.43), 208 nm (3.47). - MS (70 eV): we could never obtain the correct M^+-peak for $C_{20}H_{38}Br_2$ (438.33); evidently the molecule fragments before the molecular ion can be detected. - elemental analysis: calcd.: C 54.80, H 8.74, Br 36.46; found: C 53.17, H 8.42, Br 36.25.

Br$_2$ addition to triene **7**: According to the above procedure triene **7** (50.0 mg, 0.16 mmol) in carbon tetrachloride (10 mL) was reacted at 0 °C with a solution of bromine (360.0 mg, 2.25 mmol) in carbon tetrachloride (40 mL). Work-up according to the above protocol provided an unstable adduct **18** for which it was scarcely possible to determine the NMR spectra. - ^{1}H NMR (200.13 MHz, CDCl$_3$): δ = 1.26 (s, 36 H, *tert*-Bu), 5.71-5.78 (AA´XX´, 2 H, 4-, 7-H), 6.64-6.71 ppm (AA´XX´, 2 H, 5-, 6-H). - ^{13}C NMR (50.32 MHz, CDCl$_3$): δ = 133.88, 133.05 (d, C-4, -5, -6, -7), 96.76 (s, C-3, -8), 43.77 (s, C-2, -9, -11, -13), 31.03 ppm (q, C-1, -10, -12, -14).

Br$_2$ addition to tetraene **19**: According to the above procedure tetraene **19** (50.0 mg, 0.15 mmol) in carbon tetrachloride (10 mL) was reacted at 0 °C with a solution of bromine (12.0 mg, 0.08 mmol, 0.73 mL of a 0.1 M bromine solution) in carbon tetrachloride (20 mL); the bromine color immediately faded. Work-up after 1 h according to the above protocol and recrystallization from pentane provided a mixture (ca. 1:1) of substrate and dibromide **23**, which could not be separated further. - ^{1}H NMR (400.1 MHz, CDCl$_3$): δ = 1.24 (s, 36 H, *tert*-Bu), 5.74 (d, ^3J = 14.38 Hz, 2 H, 4-, 9-H), 6.36-6.39 (AA´XX´, 2 H, 6-, 7-H), 6.65-6.71 ppm (AA´XX´, 2 H, 5-, 8-H). - ^{13}C NMR (100.6 MHz, CDCl$_3$): δ = 134.85 (d ,C-4, -9), 133.95 (d, C-5, -8), 131.88 (d, C-6, -7), 96.59 (s, C-3, -10), 43.90 (s, C-2, -11, -13, -15), 31.06,

31.02 ppm (q, C-1, -12, -14, -16). - MS (70 eV): *m/z* (%) = 492 (0.1) [M^+], 435 (1), 411 (0.16), 355 (3), 330 (18), 297(4), 273 (08), 217 (8), 57 (100).

Br_2 addition to pentaene **20**: According to the above procedure **20** (45.0 mg, 0.13 mmol) in carbon tetrachloride (20 mL) was reacted with bromine (16 mg, 0.10 mmol, 1.0 mL of a 0.1 M solution in carbon tetrachloride, 20 mL). After 2 h at 0 °C, work-up (see above) and recrystallization from pentane afforded adduct **24** as colorless needles (28 mg, 54%), m.p. 138 °C (decomp.). - ^{1}H NMR (400.1 MHz, $CDCl_3$): δ = 1.24 (s, 36 H, *tert*-Bu), 5.74 (d, ^3J = 14.40 Hz, 2 H, 4-, 11-H), 6.32-6.33 (m, 4 H, 6-, 7-, 8-, 9-H), 6.64-6.70 ppm (m, 2 H, 5-, 10-H). - ^{13}C NMR (100.6 MHz, $CDCl_3$): δ = 134.96 (d, C-4, -11), 134.11 (d, C-5, -10), 132.58, 132.24 (d, C-6, -7, -8, -9), 96.67 (s, C-3, -12), 43.95 (s, C-2, -13, -15, -17), 31.07, 31.00 ppm (q, C-1, -14, -16, -18). - IR (KBr): nu(tilde) = 2970 (m), 1637 (C=C, w), 1393, 1370, 1362 (*tert*-Bu, m), 1006 (vs), 781 cm^{-1} (C-Br, w). - UV (acetonitrile): λ_{max} (lg ε) = 386 (4.41), 366 (4.47), 352 nm (4.41). - MS (70 eV): *m/z* (%) = 514 (<1) [M^+], 457 (1), 435 (1), 401 (<1), 377 (4), 354(12), 297 (14), 241 (18), 80 (32), 57 (100). – elemental analysis: calcd.: C 59.43, H 8.63, Br 30.41%; found: 59.45, H 8.71, Br 27.57.

Br_2 addition to tetraene **19** (with excess Br_2): According to the above procedure **19** (50.0 mg, 0.15 mmol) in carbon tetrachloride (10 mL) was reacted with bromine (150 mg, 0.94 mmol) in carbon tetrachloride (20 mL). After stirring the reaction mixture overnight at room temperature work-up as above provided the colorless solid **25** (72 mg, 73%), m.p. 110 ^{o}C (decomp.). - ^{1}H NMR (400.1 MHz, $CDCl_3$): δ = 1.25, 1.26 (2 x s, 18 H each, *tert*-Bu), 4.86-4.89 (AA´XX´, 2 H, 6-, 7-H), 5.86 (d, ^{3}J = 14.42 Hz, 2 H, 4-, 9-H), 6.23-6.29 ppm (AA´XX´, 2 H, 5-, 8-H). - ^{13}C NMR (100.6 MHz, $CDCl_3$): δ = 136.48, 132.56 (d, C-4, -5, -8, -9), 94.29 (s, C-3, -10), 56.36 (d, C-6, -7), 44.27, 43.77 (s, C-2, -11, -13, -15), 31.01, 30.90 ppm (q, C-1, -12, -14, -16). - IR (KBr): nu(tilde) = 2972 (vs), 1666 (C=C, w), 1394, 1370 (*tert*-Bu, m), 971 (s), 580 cm^{-1} (C-Br, m). - UV (acetonitrile): λ_{max} (lg ε) = 220 (4.08), 208 nm (4.16). MS (70 eV): *m/z* (%) = 433 (8), 353 (7), 271 (14), 215 (24), 57 (100). – elemental analysis: calcd.: C 43.73, H 6.57, Br 48.48%, found: C 43.95, H 6.82, Br 48.40.

Br_2 addition to pentaene **20** (with excess Br_2): According to the above procedure **20** (50.0 mg, 0.14 mmol) in carbon tetrachloride (20 mL) was reacted with bromine (120 mg, 0.70 mmol) in carbon tetrachloride (30 mL). After stirring the reaction mixture overnight at room temperature work-up

as above provided the colorless solid **26** (34 mg, 29%), m.p. 168 °C (decomp.). - ^{1}H NMR (400.1 MHz, $CDCl_3$): δ = 1.24, 1.25 (2 x s, 18 H each, *tert*-Bu), 4.29-4.30 (m, 2 H, 7-, 8-H), 5.52-5.54 (m, 2 H, 6-, 9-H), 6.02 (d, ^3J = 14.5 Hz, 2 H, 4-, 11-H), 6.33-6.39 ppm (m, 2 H, 5-, 10-H). - ^{13}C NMR (100.6 MHz, $CDCl_3$): δ = 138.26 (d, C-4, -11), 129.24 (d, C-5, -10), 94.06 (s, C-3, -12), 60.96 (d, C-7, -8), 53.52 (d, C-6, -9), 44.15, 43.25 (s, C-2, -13, -15, -17), 30.82 ppm (q, C-1, -14, -16, -18). - IR (KBr): nu(tilde) = 2963 (vs), 1654 (C=C, w),1394, 1370 (*tert*-Bu, s), 976 (s), 607 cm^{-1} (C-Br, m). - UV (acetonitrile): λ_{max} (lg ε) = 262 (3.30, sh), 200 nm (4.21, sh). - MS (70 eV): *m/z* (%) = 696 (<1), 617 (<1), 533 (1), 479 (6), 399 (6), 319 (6), 239 (12), 80 (10), 57 (100).

Br_2 addition to hexaene **21** (with excess Br_2): According to the above procedure **21** (40.0 mg, 0.11 mmol) in carbon tetrachloride (30 mL) was reacted with bromine (360 mg, 2.25 mmol) in carbon tetrachloride (30 mL). After stirring the reaction mixture overnight at room temperature work-up as above provided the colorless solid **27** (36 mg, 34%), m.p. 220 °C (decomp.). - ^{1}H NMR (400.1 MHz, $CDCl_3$): δ = 1.26-1.27 (several s, 36 H, *tert*-Bu), 4.92-4.94 (m, 2 H, 7-, 10-H), 5.02-5.07 (m, 2 H, 6-, 11-H), 5.16 (m, 2 H, 8-, 9-H), 5.91 (d, ^3J = 14.37 Hz, 2 H, 4-, 13-H), 6.25-6.33 ppm (dd, ^3J = 10.10, ^3J = 14.41 Hz, 2 H, 5-, 12-H). - ^{13}C NMR (100.6 MHz, $CDCl_3$): δ = 137.51, 137.44, 136.50, 133.06, 132.13, 132.01 (d, C-4, -5, -

12, -13), 94.20, 94.17, 94.07 (s, C-3, -14), 59.98, 59.71, 59.63, 58.93, 58.58, 57.97, 55.70, 55.58, 54.86 (d, C-6, -7, -8, -9, -10, -11), 44.42, 44.36, 44.03, 43.95 (s, C-2, -15, -17, -19), 31.33, 30.90, 29.74 ppm (q, C-1, -16, -18, -20). - IR (KBr): nu(tilde) = 2968 (vs), 1629 (C=C, w), 1386, 1370 (*tert*-Bu, m), 1139 (m), 970 (s), 602 cm^{-1} (C-Br, m). - UV (acetonitrile): λ_{max} (lg ε) = no absorption between 200 and 700 nm. - MS (70 eV): *m/z* (%) = 1020 (<1), 965 (<1), 861 (<1), 779 (4), 699 (6), 643 (8), 563 (8) 507 (8), 481 (8), 383 (100), 325 (90).

Br_2-addition to heptaene **22** (with excess Br_2): According to the above procedure **22** (50.0 mg, 0.14 mmol) in carbon tetrachloride (10 mL) was reacted with bromine (320 mg, 2.00 mmol) in carbon tetrachloride (40 mL). After stirring the reaction mixture overnight at room temp. work-up as above provided the colorless solid **28** (50 mg, 34%), m.p. 208 °C (decomp.). From the number of (partially unresolved) signals in the nmr spectra it can be concluded that different isomers have been formed in the addition. These isomers could not be separated. - ^{1}H NMR (400.1 MHz, $CDCl_3$): δ = ^{1}H NMR (400.1 MHz, $CDCl_3$): δ = 1.26 (several s, 36 H, *tert*-Bu), 4.91-4.94 (m, 2 H), 5.02-5.31 (m, 6 H), 5.84-6.02 (m, 2 H), 6.24-6.43 ppm (m, 2 H). - ^{13}C NMR (100.6 MHz, $CDCl_3$): δ = 137.41, 137.15, 136.46, 132.94, 131.98, 131.83 (d), 94.03, 93.95 (s), 60.49, 59.56, 59.42, 59.19, 59.14, 59.00, 58.89,

58.73, 58.55, 55.65, 55.61, 54.77 (d), 44.32, 44.26, 44.04, 43.93, 43.84 (s), 30.84 ppm (q). - IR (KBr): nu(tilde) = 2965 (vs), 1654 (C=C, w), 1394, 1370 (*tert*-Bu, s), 1139, 969 (s), 583 cm^{-1} (C-Br, m). - UV (acetonitrile): no absorption maxima between 200 and 700 nm - MS (70 eV): *m/z* (%) = 1044 (<1), 964 (4), 908 (18), 828 (18), 772 (22), 747 (10), 691 (26), 665 (14), 611 (28), 585 (26), 531 (28), 507 (34), 449 (28), 427 (52), 369 (40), 349 (100), 56 (100).

Epoxidations:

Epoxidation of triene **7** with *m*-chloroperbenzoic acid (MCPBA):

To a solution of triene **7** (50.0 mg, 0.16 mmol) in trichloromethane (5 mL) was added MCPBA (29.1 mg, 0.16 mmol) and the mixture stirred at room temp. overnight. For work-up the solution was treated with aqueous sodium bicarbonate solution, then washed with a saturated brine solution, and dried (sodium sulfate). The residue remaining after evaporation of the solvent was separated by silica gel column chromatography, yielding 24 mg of a 1:1-mixture of substrate and ketone **31** (ca. 25%): ^{1}H NMR (400.1 MHz, $CDCl_3$): δ = 0.97 (s, 9 H, *tert*-Bu), 1.12 (s, 9 H, *tert*-Bu), 1.34 (s, 9 H, *tert*-Bu), 1.34 (s, 9 H, *tert*-Bu), 3.47 (d, ^{3}J = 9.83 Hz, 1 H, 4-H), 5.37-5.43 (dd, ^{3}J = 9.84, ^{3}J = 14.76 Hz, 1 H, 5-H), 5.94 (d, ^{3}J = 11.21 Hz, 1 H, 7-H), 6.58-6.45 ppm (dd, ^{3}J = 11.21, ^{3}J = 14.86 Hz, 1 H, 6-H). - ^{13}C NMR (100.6 MHz, $CDCl_3$): δ = 216.80 (s, C-3), 155.44 (s, C-8), 133.91 (d, C-6), 129.98 (d, C-5), 123.07 (d, C-7), 59.34 (d, C-4), 45.57 (s, C-2), 38.61 (s, C-

9), 37.47 (s, C-13), 35.49 (s, C-11), 33.71 (q, C-14), 31.72 (q, C-10), 28.15 (q, C-12), 26.89 ppm (q, C-1). - IR (KBr): nu(tilde) = 2957 (vs), 1702 (C=O, s), 1687 (C=C, m), 1478 (m), 1392, 1366 (*tert*-Bu, m), 986 cm^{-1} (w). - UV (acetonitrile): λ_{max} (lg ε) = 254 nm (4.29). - GC/MS (70 eV): *m/z* (%) = 320 (2) [M^+], 263 (4), 235 (10), 179 (40), 151 (17), 123 (25), 109 (50), 57 (100).

Epoxidation of triene **7** with dimethyldioxirane (DMDO): Solid triene **7** (50 mg, 0.16 mmol) was placed in a 25 mL round-bottomed flask and a solution of DMDO in acetone (12.1 mL of a 0.0125 M solution) was added. After stirring at room temp. overnight, the solvent was removed in vacuo, giving colorless plates of the epoxide **32** (31 mg, 59%), m.p. 67 °C. - ^{1}H NMR (400.1 MHz, $CDCl_3$): δ = 1.20 (s, 18 H, *tert*-Bu), 1.35 (s, 18 H, *tert*-Bu), 3.69 (AA´XX´, 2 H, 5-, 6-H), 5.01 ppm (AA´XX´, 2 H, 4-, 7-H). - ^{13}C NMR (100.6 MHz, $CDCl_3$): δ = 160.66 (s, C-3, -8), 122.34 (d, C-4, -7), 59.25 (d, C-5, -6), 38.71, 37.31 (s, C-2, -9, -11, -13), 33.57 (q, C-1, -10), 31.64 ppm (q, C-12, -14). - IR (KBr): nu(tilde) = 2958 (vs), 1618 (C=C, m), 1391, 1367 (*tert*-Bu, m), 1219, 889 (epoxide, s-m). - UV (acetonitrile): λ_{max} (lg ε) = 216 nm (4.31). - MS (70 eV): *m/z* (%) = 320 (6) [M^+], 304 (2), 263 (4), 207 (22), 179 (12), 151 (10), 57 (100). - elemental analysis: calcd.: C 82.43, H 12.58; found: C 81.79, H 12.65.

Epoxidation of tetraene **19** with MCPBA: To tetraene **19** (45.0 mg, 0.14 mmol) in trichloromethane (5 mL) was added MCPBA (24.2 mg, 0.14 mmol). The mixture was stirred at room temp. overnight and worked-up as described above, giving 17 mg (36%) of **33** as a colorless oil, which decomposes on standing over longer periods of time. - ^{1}H NMR (400.1 MHz, $CDCl_3$): δ = 1.04, 1.07 (2 x s, 9 H each, *tert*-Bu), 1.20 (s, 9 H, *tert*-Bu), 1.33 (s, 9 H, *tert*-Bu), 4.35-4.38 (m, 1 H, 4-H), 5.51-5.57 (m, 2 H, 5-, 7-H), 6.00 (d, ^{3}J = 13.96 Hz, 1 H, 9-H), 5.98-6.03 (m, 1 H, 6-H), 6.74-6.81 ppm (ddd, ^{4}J = 1.04, ^{3}J = 11.20, ^{3}J = 15.02 Hz, 1 H, 8-H). - ^{13}C NMR (100.6 MHz, $CDCl_3$): δ = 156.42 (s, C-10), 136.01, 123.07 (d, C-6, -9), 132.38 (d, C-5), 130.57 (d, C-8), 127.17 (d, C-7), 81.28 (s, C-3), 81.17(d, C-4), 40.21, 40.12 (s, C-2, -13), 38.69 (s, C-11), 37.52 (s, C-15), 33.61 (q, C-16), 31.72 (q, C-12), 28.79, 28.76 ppm (q, C-1, -14). - MS (70 eV): *m/z* (%) = 346 (<1) [M^+], 289 (2), 233 (8), 205 (4), 186 (34), 57 (100).

Epoxidation of tetraene **19** with dimethyldioxirane (DMDO): Solid tetraene **19** (50 mg, 0.15 mmol) was placed in a 25 mL round-bottomed flask and a solution of DMDO in acetone (2.35 mL of a 0.0064 M solution) was added. After stirring at room temp. overnight the solvent was removed in vacuo, giving the epoxide **34** as a colorless solid of (21 mg, 49%). - ^{1}H NMR (400.1 MHz, $CDCl_3$): δ = 1.19 (s, 9 H, *tert*-Bu), 1.22 (s, 9 H, *tert*-Bu), 1.34 (s, 9 H, *tert*-Bu), 1.37 (s, 9 H, *tert*-Bu), 3.29-3.32 (dd, ^{3}J = 2.14, ^{3}J = 8.11 Hz, 1 H, 6-H), 3.74-3.76

(dd, ^3J = 2.17, ^3J = 7.18 Hz, 1 H, 5-H), 5.06 (d, ^3J = 7.23 Hz, 1 H, 4-H), 5.27-5.33 (dd, ^3J = 8.23, ^3J = 15.05 Hz, 1 H, 7-H), 6.03 (d, ^3J = 11.44 Hz, 1 H, 9-H), 7.02-7.09 ppm (dd, ^3J = 11.35, ^3J = 14.88 Hz, 1 H, 8-H). - ^{13}C NMR (100.6 MHz, $CDCl_3$): δ = 160.17 (s, C-3), 157.72 (s, C-10), 134.08 (d, C-8), 128.38 (d, C-7), 122.55 (d, C-9), 122.34 (d, C-4), 61.25 (d, C-6), 59.25 (d, C-5), 38.80 (s, C-1), 38.66 (s, C-2), 37.69 (s, C-15), 37.68 (s, C-13), 33.68 (q, C-16), 33.37 (q, C-14), 31.66 (q, C-12), 31.86 ppm (q, C-1). - IR (KBr): nu(tilde) = 3063 (w), 2954 (vs), 1619 (C=C, w), 1390, 1366 (*tert*-Bu, m), 1216, 891 (C-O-C, m and w), 987 cm^{-1} (m). - UV (acetonitrile): λ_{max} = 260 nm (qual.). - MS (70 eV): *m/z* (%) = 330 (18), 289 (8), 273 (10), 233 (10), 217 (16), 57 (100).

Epoxidation of tetraene **19** with excess DMDO: As described above tetraene **19** (50.0 mg, 0.15 mmol) was reacted with DMDO (5.6 mL of a 0.054 M solution). After work-up (see above) 50.0 mg of a colorless solid was obtained, which, according to its nmr spectra, consisted of the mono-adduct **34** and two bis-adducts to which we assign the overall structure **35** (ratio of isomers 1:1). The ratio **34**/**35** was 1:3 according to NMR spectral analysis. A chromatographic separation of these compounds failed. – Selected spectroscopic data of **35**: ^{1}H NMR (400.1 MHz, $CDCl_3$): δ = 1.18 (s, 18 H, *tert*-Bu), 1.36, 1.37 (2 x s, 18 H, *tert*-Bu), 2.88, 2.93 (ps-s, 2 H, 6-, 7-H), 3.85 (d, ^3J = 7.29 Hz, 2 H, 5-, 8-H), 4.98 (d, ^3J = 7.63 Hz) and 4.99 ppm (^3J = 7.54 Hz, 2 H, 4-, 9-H). – Isomer 1: ^{13}C NMR (100.6

MHz, $CDCl_3$): δ = 161.41 (s, C-3, -10), 121.44 (d, C-4, -9), 58.46 (d, C-6, -7), 54.24 (d, C-5, -8), 38.78 (s, C-2, -11), 37.44 (s, C-13, -15), 33.45 (q, C-14, -16), 31.60 ppm (q, C-1, -12). – Isomer 2: ^{13}C NMR (100.6 MHz, $CDCl_3$): δ = 161.06 (s, C-3, -10), 121.71 (d, C-4, -9), 59.16 (d, C-6, -7), 55.28 (d, C-5, -8), 38.75 (s, C-2, -11), 37.42 (s, C-13, -15), 33.45 (q, C-14, -16), 31.60 (q, C-1, -12). - IR (KBr): nu(tilde) = 2958 (vs), 1619 (C=C, w), 1389, 1368 (*tert*-Bu, m), 1218 (m), 890 (s), 805 cm^{-1} (w). - UV (acetonitrile): λ_{max} (lg ε) = 206 nm (4.35). - MS (70 eV): *m/z* (%) = 380 (18) [M^+ + Na^+], 364 (16), 347 (26), 331 (20), 291 (54), 274 (56), 233 (68), 142 (100), 125 (84).

Diels-Alder-additions; *Reactions with N-phenyltriazolinedione* (**36**, PTAD):

Attempted addition of **36** to triene **7**: To a solution of **7** (50.0 mg, 0.16 mmol) in anhydrous THF (5 mL) was added **36** (28.7 mg, 0.16 mmol) and the mixture kept at room temp. for 24 h. The solvent was removed in vacuo and the solid residue chromatographed on silica gel (dichloromethane/pentane = 1:3): 25 mg (50%) of **7** was recovered, together with polymeric material.

Addition of **36** to tetraene **19**: According to the above procedure tetraene **19** (50.0 mg, 0.15 mmol) was reacted with **36** (26.5 mg, 0.15 mmol) in THF (5 mL, room temp., 12 h). Work-up yielded 57 mg (75%) of the adduct **37**, which was recrystallized from diethyl ether/pentane: colorless needles, m.p. 163 °C. -

^{1}H NMR (400.1 MHz, $CDCl_3$): δ = 1.21 (s, 18 H, *tert*-Bu), 1.40 (s, 18 H, *tert*-Bu), 5.23 (d, ^3J = 9.28 Hz, 2 H, 4-, 9-H), 5.55 (AA´XX´, 2 H, 5-, 8-H), 5.84 (AA´XX´, 2 H, 6-, 7-H), 7.41-7.49 ppm (m, 5 H, phenyl). - ^{13}C NMR (100.6 MHz, $CDCl_3$): δ = 156.33 (s, C-3, -10), 152.06 (s, C-17, -18), 131.29 (s, C-19), 129.08, 125.66 (d, C-20, -21), 127.87 (d, C-22), 124.64 (d, C-6, -7), 121.19 (d, C-4, -9), 54.73 (d, C-5, -8), 39.06 (s, C-2, -15), 37.48 (s, C-11, -13), 33.65 (q, C-12, -14), 31.82 ppm (q, C-1, -16). - IR (KBr): nu(tilde) = 3016 (m, CH-phenyl), 2961 (m), 1723 (vs, C=O), 1711 (vs, imide, vs), 1602 (m, C=C), 1369, 1312 (m, *tert*-Bu), 760 cm^{-1} (m). - UV (acetonitrile): λ_{max} (lg ε) = 216 nm (4.57). - MS (70 eV): *m/z* (%) = 505 (18) [M^+], 448 (40), 273 (100), 57 (38). - HRMS: $C_{32}H_{47}N_3O_2$ (505.75): calcd. 505.366, found 505.366 ±2 ppm. – elemental analysis: calcd.: C 76.00, H 9.37, N 8.31; found: C 75.89, H 9.41, N 8.07.

Addition of **36** to pentaene **20**: According to the above procedure pentaene **20** (50.0 mg, 0.14 mmol) was reacted with **36** (24.5 mg, 0.14 mmol) in THF (5 mL, room temp., 2 h). Work-up yielded 49 mg (66%) of the adduct **38**: colorless powder, m.p. 171 °C. - ^{1}H NMR (400.1 MHz, $CDCl_3$): δ = 1.19 (s, 9 H, *tert*-Bu), 1.22 (s, 9 H, *tert*-Bu), 1.36 (s, 9 H, *tert*-Bu), 1.40 (s, 9 H, *tert*-Bu), 4.94-4.98 (m, 1 H, 8-H), 5.24 (d, ^3J = 9.54 Hz, 1 H, 4-H), 5.54-5.63 (m, 2 H, 5-, 9-H), 5.77-5.81 (ddd, J = 1.71, J = 3.54, ^3J = 10.25 Hz, 1 H, 7-H), 5.89-5.93 (ddd, J = 1.94, J = 3.71, ^3J = 10.25 Hz, 1 H, 6-H), 6.04 (d, ^3J = 11.17

Hz, 1 H, 11-H), 6.98-7.05 (ddd, ^4J = 0.55, ^3J = 11.21, ^3J = 14.87 Hz, 1 H, 10-H), 7.30-7.34 (m, 1 H, 22-H), 7.41-7.45 (m, 2 H, 22-H), 7.49-7.52 ppm (m, 2 H, 22-H). - ^{13}C NMR (100.6 MHz, $CDCl_3$): δ = 158.84, 156.69, 152.72, 151.77, 131.36 (s, C-3, -12, -19, -20, -21), 134.07 (d, C-10), 129.09, 127.91 (d, C-22), 126.55 (d, C-9), 125.57 (d, C-22), 125.32 (d, C-6), 124.49 (d, C-7), 122.33 (d, C-11), 120.54 (d, C-4), 56.52 (s, C-8), 54.76 (d, C-5), 39.18, 38.95, 37.79, 37.58 (s, C-2, -13, -15, -17), 33.87 (q, C-14), 33.75 (q, C-16), 31.96 (q, C-1), 31.70 ppm (q, C-18). - IR (KBr): nu(tilde) = 3010 (m), 2954 (m), 1723 (C=O, vs), 1654 (C=C, m), 1391, 1368 (*tert*-Bu, s), 1215 (m), 978 (m), 760 cm^{-1} (m). - UV (aceto-nitrile): λ_{max} (lg ε) = 254 (4.44), 220 (4.43), 194 nm (4.69). - MS (70 eV): *m/z* (%) = 531 (16) [M^+], 474 (8), 418 (12), 299 (62), 241 (42), 57 (100). - HRMS: $C_{34}H_{49}N_3O_2$ (531.34): calcd.: 531.382, found: 531.381 ±2 ppm. – elemental analysis: calcd.: C 76.79, H 9.29, N 7.90; found: C 76.36, H 9.46, N 7.52.

Addition of **36** to hexaene **21**: According to the above procedure hexaene **21** (40.0 mg, 0.11 mmol) was reacted with **36** (22.9 mg, 0.13 mmol) in THF (5 mL, room temp., 1 h). Work-up (recrystallization from pentane) yielded 14 mg (24%) of the adduct **39**: colorless powder, m.p. 176 °C. – ^{1}H NMR (400.1 MHz, $CDCl_3$): δ = 1.21 (s, 18 H, *tert*-Bu), 1.36 (s, 18 H, *tert*-Bu), 5.00 (AA´XX´, 2 H, 7-, 10-H), 5.53-5.59 (dd, ^3J = 7.30 Hz, ^3J = 14.6 Hz, 2 H, 6-, 11-H), 5.89 (AA´XX´, 2 H, 8-, 9-H), 6.00 (d, ^3J = 11.30 Hz, 2 H, 4-, 13-H), 6.97-7.04 (dd, ^3J = 11.24, ^3J =

14.81 Hz, 2 H, 5-, 12-H), 7.31-7.34 (m, 1 H, 26-H), 7.41-7.50, 7.51-7.53 ppm (m, 2 H each, 24-, 25-H). - ^{13}C NMR (100.6 MHz, $CDCl_3$): δ = 158.88, 151.88, 151.37 (s, C-3, -14, -21, -22, -23), 134.03 (d, C-5, -12), 129.00, 125.43 (d, C-24, -25), 126.17 (d, C-26), 125.52 (d, C-6, -11), 124.86 (d, C-8, -9), 122.31 (d, C-4, -13), 56.19 (d, C-7, -10), 38.94, 37.79 (s, C-2, -15, -17, -19), 33.86 (q, C-16, -18), 31.68 ppm (q, C-1, -20). - IR (KBr): nu(tilde) = 3003 (w), 2956 (m), 1720 (C=O, vs), 1687 (C=C, w), 1367 (*tert*-Bu, m), 977 (w) 755 cm^{-1} (w). - UV (acetonitrile): λ_{max} (lg ε) = 298 (4.05), 256 nm (4.66). - MS (70 eV): *m/z* (%) = 558 (10) [M^+], 501 (8), 444 (9), 366 (32), 324 (34), 267 (42), 57 (100). - HRMS: $C_{36}H_{51}N_3O_2$ (557.82): calcd.: 557.398, found 557.397 ±2 ppm

<u>Double addition product **40** by addition of excess **36** to hexaene **21**:</u> As above **36** (45.0 mg, 0.257 mmol) was added to a solution of hexaene **21** (40.0 mg, 0.11 mmol) in THF (5 mL) and the reaction mixture stirred overnight at room temp. After work-up (recrystallization from pentane) the bis-adduct **40** (36 mg, 47%) was obtained as a colorless solid, m.p. 238 °C. - ^{1}H NMR (400.1 MHz, $CDCl_3$): δ = 1.12 (s, 18 H, *tert*-Bu), 1.34 (s, 18 H, *tert*-Bu), 5.13 (m, 2 H, 8-, 9-H), 5.23 (d, ^3J = 9.43 Hz, 2 H, 4-, 13-H), 5.54-5.56 (m, 2 H, 5-, 12-H), 5.98-6.04 (m, 4 H, 6-, 7-, 10-11-H), 7.30-7.34 (m, 2 H, 26-, 32-H), 7.40-7.44 (m, 4 H, 25-, 31-H), 7.52-7.54 ppm (m, 4 H, 4-, 30-H). - ^{13}C NMR (100.6 MHz, $CDCl_3$): δ = 156.36 (s, C-3, -19), 152.84, 150.45 (s, C-21, -22, -27, -28), 131.30 (s, C-23, -29), 129.02 (d, C-

25, -31), 128.02 (d, C-26, -32), 126.16 (d, C-24, -30), 128.84, 119.44 (d, C-6, -7, -10, -11), 121.25 (d, C-4, -13), 55.69 (d, C-5, -12), 52.22 (d, C-8, -9), 39.11 (s, C-17, -19), 37.51 (s, C-2, -25), 33.63 (q, C-1, -16), 31.74 ppm (q, C-18, -20). - IR (KBr): nu(tilde) = 3004 (w), 2956 (m), 1717 (C=O, vs), 1601 (C=C, w), 1370, 1326 (*tert*-Bu, w), 791 (w), 764 cm^{-1} (m). - UV (acetonitrile): λ_{max} (lg ε) = 194 (4.94), 210 nm (4.62, sh). - MS (70 eV): *m/z* (%) = 732 (3) [M^+], 676 (<1), 557 (<1), 500 (<1), 366 (83), 310 (100), 133 (46), 57 (68). - HRMS: $C_{44}H_{56}N_6O_4$(732.96): calcd.: 732.436, found 732.435 ±2 ppm. – elemental analysis: calcd.: C 72.10, H 7.70 N 11.47, found: C 69.08, H 7.52, N 11.25.

<u>Double addition product **41** by addition of excess **36** to heptaene **22**:</u> As above dienophile **36** (12.9 mg, 0.07 mmol) was added to a solution of heptaene **22** (30.0 mg, 0.07 mmol) in THF (10 mL). The reaction mixture was stirred overnight, the solvent was removed in vacuo, and the residue was separated by silica gel column chromatography with petroleum ether/diethyl ether (3:1). As shown by GC/MS analysis the first fraction (10 mg, 25%) consisted of a mono-addition product, and the second of a bis-adduct of **22**, to which we assign structure **41.** The stoichiometry of the adducts was determined by mass spectrometric analysis. - Monoadduct: MS (70 eV): *m/z* (%) = 583 (10) [M^+], 526 (8), 366 (20), 310 (30), 57 (100). – Bis-adduct **41:** MS (70 eV): *m/z* (%) = 758 (12) [M^+], 701 (6), 392 (30), 366 (82), 310 (100).

Reactions with tetracyanoethylene (TCNE):

Addition of TCNE to the tetraene **19**: A solution of tetraene **19** (50.0 mg, 0.15 mmol) and TCNE (19.6 mg, 0.15 mmol) in anhydrous THF (5 mL) was heated for 8 h at 66 °C. For work-up, the solvent was removed by rotary evaporation and the residue was separated by silica gel chromatography with petroleum ether/diethyl ether (9:1). The first fraction (20 mg) consisted of starting material **19**, the second fraction of the mono-adduct **43** (29 mg, 70%), colorless solid, m.p. ca. 170 °C (decomp.). - ^{1}H NMR (400.1 MHz, $CDCl_3$): δ = 1.28 (s, 18 H, *tert*-Bu), 1.40 (s, 18 H, tert-Bu), 4.47 (AA´XX´, 2 H, 5-, 8-H), 5.58 (d, ^{3}J = 11.52 Hz, 2 H, 4-, 9-H), 5.74 ppm (AA´XX´, 2 H, 6-, 7-H). - ^{13}C NMR (100.6 MHz, $CDCl_3$): δ = 163.63 (s, C-3, -10), 126.07 (d, C-6, -7), 117.60 (d, C-4, -9), 111.56, 110.31 (s, C-19, -20, -21, -22), 42.78 (s, C-17, -18), 41.93 (d, C-5, -8), 37.65, 33.53 (s, C-2, -11, -13, -15), 33.75 (q, C-12, -14), 31.59 ppm (q, C-1, -16). - MS (70 eV): *m/z* (%) = 458 (1) [M^+], 401 (2), 330 (26), 273 (24), 217 (22), 57 (100). - HRMS: $C_{30}H_{42}N_4$ (458.69): calcd.: 458.341, found 458.340 ±2 ppm.

Addition of TCNE to the pentaene **20**: Following the above procedure, a solution of the pentaene **20** (50.0 mg, 0.14 mmol) and TNCE (18.0 mg, 0.14 mmol) in THF (2 mL) was kept at room temp. After 2 h the yellow color of **20** had vanished. The mixture was kept at room temp. overnight and worked-up by removal of the solvent in vacuo. Silica gel column chromatography (petroleum ether/diethyl ether = 19:1)

furnished **44** as a colorless solid (52 mg, 77%), m.p. 187-188 °C. - ^{1}H NMR (400.1 MHz, $CDCl_3$): δ = 1.25 (s, 9 H, *tert*-Bu), 1.27 (s, 9 H, *tert*-Bu), 1.40 (s, 9 H, tert-Bu), 1.41 (s, 9 H, *tert*-Bu), 3.73-3.75 (d, *J* = 8.88 Hz, 1 H, 7-H), 4.49-4.52 (d, *J* = 11.88 Hz, 1 H, 10-H), 5.54-5.57 (d, ^3J = 8.83 Hz, 1 H, 6-H), 5.58 (d, ^3J = 11.73 Hz, 1 H, 11-H), 5.79-5.82 (m, 2 H, 8-, 9-H), 6.06 (d, ^3J = 11.25 Hz, 1 H, 4-H), 7.14-7.20 ppm (dd, ^3J = 11.22, ^3J = 14.27 Hz, 1 H, 5-H). - ^{13}C NMR (100.6 MHz, $CDCl_3$): δ = 163.71, 162.04 (s, C-3, -12), 138.97 (d, C-5), 126.20, 124.77 (d, C-8, -9), 122.16 (d, C-6), 121.46 (d, C-4), 117.24 (d, C-11), 111.54, 111.51, 110.06 (s, C-21, -22, -23, -24), 45.10 (d, C-7), 42.51 (d, C-10), 42.44 (s, C-19, -20), 40.18, 39.11, 37.92, 37.66 (s, C-2, -13, -15, -17), 33.93 (q, C-16), 33.81 (q, C-14), 31.58 (q, C-18), 31.52 ppm (q, C-1). - IR (KBr): nu(tilde) = 2962 (vs), 2254 (w), 1633 (C=C, m), 1394, 1369 (*tert*-Bu, m), 1217 (m), 984 (m). - UV (acetonitrile): λ_{max} (lg ε) = 262 (4.43), 218 (4.19), 198 nm (4.18). - MS (70 eV): *m/z* (%) = 484 (1) [M^+], 427 (10), 371 (12), 299 (30), 57 (100). - elemental analysis: calcd.: C 79.29, H 9.15, N 11.56; found: C 79.20, H 9.31, N 11.45.

Addition of TCNE to the hexaene **21**: As above, a solution of hexaene **21** (40.0 mg, 0.11 mmol) and TCNE (16.8 mg, 0.13 mmol) in THF (5 mL) was kept at room temp. After 2 h the solvent was removed by rotary evaporation and the residue purified by silica gel column chromatography with petroleum ether/diethyl ether (19:1). After recrystallization from pentane colorless

needles of the mono-adduct **45** (26.0 mg, 49%) were obtained, m.p. 221 °C (decomp.). - ^{1}H NMR (400.1 MHz, $CDCl_3$): δ = 1.24 (s, 18 H, *tert*-Bu), 1.39 (s, 18 H, *tert*-Bu), 3.80 (AA´XX´, 2 H, 7-, 10-H), 5.55-5.61 (dd, ^{3}J = 8.69, ^{3}J = 14.75 Hz, 2 H, 6-, 11-H), 5.91 (AA´XX´, 2 H, 8-, 9-H), 6.04 (d, ^{3}J = 11.27 Hz, 2 H, 4-, 13-H), 7.12-7.18 ppm (dd, ^{3}J = 11.32, ^{3}J = 14.59 Hz, 2 H, 5-, 12-H). - ^{13}C NMR (100.6 MHz, $CDCl_3$): δ = 162.21 (s, C-3, -14), 139.19 (d, C-5, -12), 125.45 (d, C-8, -9), 121.88 (d, C-6, -11), 121.52 (d, C-4, -13), 111.51, 109.75 (s, C-23, -24, -25, -26), 45.74 (d, C-7, -10), 42.39 (s, C-21, -22), 39.19, 37.99 (s, C-2, -15, -17, -19), 34.02 (q, C-16, -18), 31.59 ppm (q, C-1, -20). - IR (KBr): nu(tilde) = 2959 (vs), 2248 (-CN, w), 1627 (C=C, m),1393, 1367 (*tert*-Bu, m), 977 cm^{-1} (m). - UV (acetonitrile): λ_{max} (lg ε) = 266 nm (4.70). - MS (70 eV): *m/z* (%) = 510 (2) [M^+], 453 (8), 397 (16), 325 (16), 258 (12), 57 (100). - HRMS: $C_{34}H_{46}N_4$ (510.40): calcd.: 510.372; found 510.372 ±2 ppm. – elemental analysis: calcd.: C 79.95, H 9.08, N 10.97, found: C 79.75, H 9.30, N 10.95.

Addition of TCNE to the heptaene **22**: As above, a solution of heptaene **22** (103.0 mg, 0.25 mmol) and TCNE (30.0 mg, 0.25 mmol) in THF (10 mL) was kept at room temp. After storing at room temp. overnight, the solvent was removed by rotary evaporation and the residue purified by silica gel column chromatography with petroleum ether/diethyl ether (8:2): 100.0 mg (92%) of a slightly yellow solid, **46**, m.p. 135 °C (decomp.). - ^{1}H NMR (400.1 MHz, $CDCl_3$): δ = 1.23, 1.24, 1.38, 1.39 (4 x s,

9 H each, *tert*-Bu), 3.77-3.79 (m, 2 H, 9-, 12-H), 5.54-5.60 (dd, ^3J = 8.95, ^3J = 14.53 Hz, 1 H, 13-H), 5.70-5.76 (dd, ^3J = 8.66, ^3J = 14.91 Hz, 1 H, 8-H), 5.88-5.94 (m, 2 H, 10-, 11-H), 6.05 (d, ^3J = 11.18 Hz, 1 H, 15-H), 6.07 (d, ^3J = 11.56 Hz, 1 H, 4-H), 6.11-6.17 (dd, ^3J = 10.73, ^3J = 14.55 Hz, 1 H, 6-H), 6.54-6.60 (dd, ^3J = 10.70, ^3J = 14.96 Hz, 1 H, 7-H), 6.97-7.03 (dd, ^3J = 11.63, ^3J = 14.43 Hz, 1 H, 5-H), 7.13-7.19 ppm (dd, ^3J = 11.23, ^3J = 14.53 Hz, 1 H, 14-H). - ^{13}C NMR (100.6 MHz, $CDCl_3$): δ = 162.12, 160.70 (s, C-3, -16), 140.33 (d, C-7), 139.15 (d, C-14), 136.59 (d, C-5), 129.04 (d, C-6), 125.54, 125.16 (d, C-10, -11), 123.19(d, C-4), 121.74 (d, C-13), 121.57 (d, C-8), 121.43 (d, C-15), 111.40, 111.38, 109.68, 109.61 (s, C-25, -26, -27, -28), 45.54, 45.44 (d, C-9, -12), 42.45, 42.18 (s, C-23, -24), 39.11, 37.98, 37.92 (s, C-2, -17, -19, - 21), 33.95, 33.84, 31.63, 31.52 ppm (q, C-1, -18, -20, -22). - IR (KBr): nu(tilde) = 2958 (vs), 2256 (-CN, w), 1216 (C=C, m), 994 (s). - UV (acetonitrile): λ_{max} (lg ε) = 296 (4.65), 272 nm (4.52). - MS (70eV): *m/z* (%) = 536 (4) [M^+], 479 (10), 422 (18), 57 (100). - HRMS: $C_{36}H_{48}N_4$ (536.80): calcd.: 536.387, found: 536.387 ±2 ppm. - Elemental analysis: calcd.: C 80.55, H 9.01, N 10.44%; found C 80.58, H 9.36, N 9.74.

Addition of TCNE to the nonaene **42**: As above, a solution of nonaene **42** (50.0 mg, 0.11 mmol) and TCNE (27.8 mg, 0.22 mmol) in THF (5 mL) was kept at room temp. After 12 h the solvent was removed by rotary evaporation and the residue purified by silica gel column chromatography with petroleum ether/diethyl

ether (8.5:1.5). Three fractions were obtained: fraction 1, 6 mg (9%), slightly yellow solid, m.p. 171 °C (decomp.), mono-adduct **47**; fraction 2: 15 mg (18%), colorless solid, m.p. 212 °C (decomp.), bis-adduct **48**; fraction 3: black residue (20 mg), which could not be identified. – Spectroscopic data of **47**: ^{1}H NMR (400.1 MHz, $CDCl_3$): δ = 1.231, 1.233 (s, 9 H each, *tert*-Bu), 1.38 (s, 18 H, *tert*-Bu), 3.75-3.77 (m, 2 H, 11-, 14-H), 5.68-5.74 (m, 2 H, 10-, 15-H), 5.91 (m, 2 H, 12-, 13-H), 6.07 (d, ^3J = 11.58 Hz, 1 H, 19-H), 6.08 (d, ^3J = 11.56 Hz, 1 H, 4-H), 6.11-6.17 (m, 2 H, 6-, 17-H), 6.20-6.27 (dd, ^3J = 10.65, ^3J = 14.86 Hz, 1 H, 8-H), 6.46-6.63 (m, 3 H, 7-, 9-, 16-H), 6.92-6.98 (m, 1 H, 5-H), 6.98-7.04 ppm (m, 1 H, 18-H). - ^{13}C NMR (100.6 MHz, $CDCl_3$): δ = 160.85, 159.60 (s, C-3, -20), 140.37, 140.05, 137.95, 136.69, 135.54, 130.98, 129.06, 128.67, 125.40, 125.34, 123.70, 123.22, 121.87, 121.53 (d, C-4, -4, -5, -6, -7, -8, -9, -10, -11, -12, -13, -14, -15, -16, -17, -18, -19), 111.10, 109.63 (s, C-29, -30, -31, -32), 45.47, 45.46 (d, C-11, -14), 42.28, 39.17, 38.05, 38.04 (s, C-2, -21, -23, -25), 33.89, 33.85, 31.73, 31.69 ppm (q, C-1, -22, -24, -26). - IR (KBr): nu(tilde) = 2958 (2253 (-CN, vw), 1632 (C=C, m), 1392, 1367 (*tert*-Bu, m), 1218 (m), 998 cm^{-1} (vs). - UV (acetonitrile): λ_{max} (lg ε) = 312 (4.83), 322 (4.84), 334 nm (4.77, sh). - MS (70 eV): *m/z* (%) = 588 (8) [M^+], 531 (16), 475 (16), 460 (100), 403 (14), 346 (18), 128 (18), 57 (37). - HRMS: $C_{40}H_{52}N_4$ (588.88): calcd.: 588.419, found: 588.418 ±2 ppm. – Spectroscopic data of **48**: ^{1}H NMR (400.1 MHz, $CDCl_3$): δ = 1.26

(s, 18 H, *tert*-Bu), 1.40 (s, 18 H, *tert*-Bu), 3.81-3.85 (m, 4 H, 7-, 10-, 13-, 16-H), 5.50-5.56 (dd, ^3J = 8.73, ^3J = 14.54 Hz, 2 H, 6-, 17-H), 6.01 (m, 4 H, 8-, 9- 14-, 15-H), 6.07 (d, ^3J = 11.27 Hz, 2 H, 4-, 19-H), 6.22-6.24 (AA´XX´, 2 H, 11-, 12-H), 7.18-7.24 (dd, ^3J = 11.20, ^3J = 14.49 Hz, 2 H, 5-, 18-H). - ^{13}C NMR (100.6 MHz, $CDCl_3$): δ = 162.86 (s, C-3, -20), 139.68, 133.08, 127.25, 123.37, 121.36, 120.94 (d, C-4, -5, -6, -8, -9, -11, -12, 14, -15, -17, -18, -19), 110.66, 110.00 (s, C-29, -30, -31, -33, -35, -36, -37, -38), 45.32, 44.48 (d, C-7, -10, -13, -16), 42.13, 41.91, 39.28, 38.07 (s, C-2, -21, -23, -25, -27, -28, -33, -34), 34.06, 31.60 ppm (q, C-1, -22, -24, -26). - IR (KBr): nu(tilde) = 2960 (vs), 2254 (-CN, vw), 1631 (C=C, m), 1393, 1369 (*tert*-Bu, m), 1217 (m), 981 cm^{-1} (m). - UV (acetonitrile): λ_{max} (lg ε) = 262 (4.66), 208 (4.39), 196 nm (4.37). - MS (70 eV): *m/z* (%) = 716 (2) [M^+], 659 (12), 586 (9), 529 (50), 473 (42), 460 (54), 403 (15), 346 (15), 128 (52), 57 (100). - HRMS: $C_{46}H_{52}N_8$ (716.97): calcd.: 716.431, found 716.430 ±2 ppm.

Irradiation of 19: A solution of tetraene **19** (100 mg, 0.30 mmol) in deuteriochloroform (5 mL) was irradiated with a 450 W sun lamp in an open NMR tube (with air access). After 9 h 93% of the starting material had been consumed (NMR analysis). The product was purified by silica gel chromatography with petroleum either/diethyl ether (9:1): 47 mg (46%) of the colorless *endo*-peroxide **52**, m.p. 89 °C. - ^{1}H NMR (400.1 MHz, $CDCl_3$): δ = 1.21 (s, 18 H, *tert*-Bu), 1.34 (s, 18 H, *tert*-Bu),

5.34 (d, ^3J = 9.79 Hz, 2 H, 4-, 9-H), 5.62 (AA´XX, 2 H, 5-, 8-H), 5.93 ppm (AA´XX´, 2 H, 6-, 7-H). - ^{13}C NMR (100.6 MHz, $CDCl_3$): δ = 159.08 (s, C-3, -10), 127.23 (d, C-6, -7), 120.88 (d, C-4, -9), 76.17 (d, C-5, -8), 38.94 (s, C-2, -15), 37.55 (s, C-11, -13), 33.99 (q, C-12, -14), 31.64 ppm (q, C-1, -16). - IR (KBr): nu(tilde) = 2961 (vs), 1618 (C=C, w), 1469 (m), 1488 (m), 1391, 1365 (*tert*-Bu, m), 993 (m), 734 cm^{-1} (m). - UV (acetonitrile): λ_{max} (lg ε) = 212 nm (4.40). - MS (70 eV): *m/z* (%) = 362 (<1) [M^+], 346 (2), 330 (100), 273(34), 217 (37), 189 (37), 109 (28), 57 (87). – elemental analysis: calcd.: C 79.50, H 11.68; found: C 79.78, H 12.14.

X-ray structure determinations

All compounds crystallized solvent-free. Crystals were mounted in inert oil on glass fibres and transferred to the cold gas stream of the diffractometer. Intensity data were recorded using monochromated Mo Kα radiation (λ = 0.71073 Å). Absorption corrections were performed for **24** on the basis of multi-scans; other datasets were not corrected. The structures were refined anisotropically on F^2 using the program SHELXL-97 [2]. Hydrogens were refined using rigid methyl groups allowed to rotate but not tip, or a riding model starting from calculated positions. *Special features*: Although we are confident that the structure of **4** is at least qualitatively correct, the large difference peaks of 1 e Å^{-3}

are unsatisfactory. They probably correspond to a small amount of contamination by the corresponding diol, a synthetic precursor of **4**, which has cell constants similar to those of **4** [3] and therefore may be difficult to remove completely. Crystals of compound **37** could not be measured below -70°C because they shattered, presumably because of thermal stress and/or a phase change.

Data for compound **4** were recorded by the late Dr. Peter Bubenitschek. Crystallographic data have been deposited with the Cambridge Crystallographic Data Centre as supplementary publications no. CCDC-1400892 (**4**), -1062595 (**24**), -1062596 (**37**), -1062594 (**42**), -1062593 (**524**). Copies of the data can be obtained free of charge from www.ccdc.cam.ac.uk/data_request/cif.

Table 1. Crystallographic data.

Compound	**4**	**24**	**37**	**42**	**52**
Formula	$C_{20}H_{40}$	$C_{26}H_{44}Br_2$	$C_{32}H_{47}N_3O_2$	$C_{30}H_{42}N_4$	$C_{24}H_{42}O_2$
M_r	280.52	516.43	505.73	458.68	362.58
Habit	colourless prism	colourless needle	colourless prism	colourless prism	colourless tablet
Cryst. size (mm)	0.8 × 0.4 × 0.3	0.4 × 0.14 × 0.12	0.9 × 0.45 × 0.3	0.6 × 0.4 × 0.2	0.4 × 0.2 × 0.13
Crystal system	triclinic	monoclinic	triclinic	triclinic	triclinic
Space group	$P\bar{1}$	$P2_1/n$	$P\bar{1}$	$P\bar{1}$	$P\bar{1}$
Temperature (°C)	–130	–130	–70	–100	–130
Cell constants:					
a (Å)	8.493(3)	6.4430(8)	11.886(3)	14.501(3)	6.2952(8)
b (Å)	11.383(4)	14.0523(14)	12.682(2)	15.155(3)	12.8507(16)
c (Å)	11.894(4)	14.8513(15)	21.281(4)	15.548(3)	15.156(2)
α (°)	102.87(2)	90	83.658(10)	101.11(2)	105.132(5)
β (°)	104.22(2)	99.124(6)	74.666(16)	117.48(2)	95.670(5)
γ (°)	111.42(2)	90	76.382(18)	99.96(2)	96.319(5)
V (Å^3)	973.5	1327.6	3002.6	2836.7	1165.9
Z	2	2	4	4	2
D_x (Mg m^{-3})	0.957	1.292	1.119	1.074	1.033
μ (mm^{-1})	0.05	3.1	0.07	0.06	0.06
F(000)	320	540	1104	1000	404
Diffractometer	Stoe STADI-4	Siemens SMART 1000 CCD	Siemens P4	Siemens P4	Siemens SMART 1000 CCD
$2\theta_{max}$	50	57	50	50	56.6
Refl. measured	3643	14045	13098	9809	18228
Refl. indep.	3430	3371	10496	9756	5760
R_{int}	0.014	0.048	0.025	0.043	0.088
Transmissions	no abs. corr.	0.634–0.983	no abs. corr.	no abs. corr.	no abs. corr.
Parameters	193	133	691	636	247
Restraints	0	0	0	0	0
$wR(F^2$, all refl.)	0.176	0.070	0.133	0.143	0.128
$R(F, >4s(F))$	0.062	0.029	0.050	0.061	0.049
S	1.02	0.95	0.95	0.88	1.02
max. Δρ (e Å^{-3})	0.99	0.73	0.33	0.20	0.31

References Supporting Information:

[1] Adam, W.; Bialas, J.; Hadjiarapoglou, L. Chem. Ber. **1991**, *124*, 2377-2377.

[2] Sheldrick, G. M. Acta Cryst. **2008**, *A64*, 112-122.

[3] Hänel, R.; Bubenitschek, P.; Hopf, H.; Jones, P. G. 2006. Private communication to the Cambridge Crystallographic Data Centre, refcode IDEPUD.
